# First principles-based design of lightweight high entropy alloys

**DOI:** 10.1038/s41598-023-49258-z

**Published:** 2023-12-18

**Authors:** Viacheslav Sorkin, Zhi Gen Yu, Shuai Chen, Teck Leong Tan, Zachary Aitken, Yong-Wei Zhang

**Affiliations:** 1 Institute of High Performance Computing (IHPC), Agency for Science, Technology and Research (A*STAR), 1 Fusionopolis Way, #16-16 Connexis, Singapore, 138632 Republic of Singapore; 2 Materials Genome Institute, Shanghai University, Shanghai, 200444 China

**Keywords:** Atomistic models, Computational methods

## Abstract

Recently, the design of lightweight high entropy alloys (HEAs) with a mass density lower than 5 g/cm^3^ has attracted much research interest in structural materials. We applied a first principles-based high-throughput method to design lightweight HEAs in single solid-solution phase. Three lightweight quinary HEA families were studied: AlBeMgTiLi, AlBeMgTiSi and AlBeMgTiCu. By comprehensively exploring their entire compositional spaces, we identified the most promising compositions according to the following design criteria: the highest stability, lowest mass density, largest elastic modulus and specific stiffness, along with highest Pugh’s ratio. We found that HEAs with the topmost compositions exhibit a negative formation energy, a low density and high specific Young’s modulus, but a low Pugh’s ratio. Importantly, we show that the most stable composition, Al_0.31_Be_0.15_Mg_0.14_Ti_0.05_Si_0.35_ is energetically more stable than its metallic compounds and it significantly outperforms the current lightweight engineering alloys such as the 7075 Al alloy. These results suggest that the designed lightweight HEAs can be energetically more stable, lighter, and stiffer but slightly less ductile compared to existing Al alloys. Similar conclusions can be also drawn for the AlBeMgTiLi and AlBeMgTiCu. Our design methodology and findings serve as a valuable tool and guidance for the experimental development of lightweight HEAs.

## Introduction

High entropy alloys (HEAs)^1–4^, which include multiple principal elements, have attracted substantial research attention due to their exceptional mechanical properties, such as high fracture toughness, hardness, fatigue and corrosion resistances, yield strength, ductility and thermal stability^1,2,5,6^. In many applications, HEAs can significantly outperform traditional and super-alloys^2^, especially at low and high temperatures^3,4^. Recently, the design of lightweight HEAs (with a density lower than 5 g/cc) has become one of the hottest research topics in metallurgy. In view of the outstanding performance of HEAs, the lightweight HEAs may have superior performance than traditional lightweight materials such as Al, Mg, and Ti alloys, with a large number of potential industry applications^7^. Yet the chemical and compositional design space of HEAs is exceptionally vast, which makes the use of the traditional experimental methods for HEA screening an overwhelming task. Thus, an efficient, accurate strategy is required for the design of lightweight HEAs for specific applications^8,9^.

Different computational methods, such as CALPHAD^10,11^, molecular dynamics (MD)^12–14^ and density functional theory (DFT)^15,16^, have been applied for the HEA design. Lately, machine learning (ML) based approach for the HEA design is also gaining momentum^17–19^. For example, Senkov et al.^6,9,20^ developed a CALPHAD-based strategy for the design of equimolar HEAs in a single solid-solution phase at elevated temperatures, which allows rapid screening of HEAs. The method was further developed^11,21,22^ to be used for high-throughput (HT) design of advanced lightweight HEAs for high-temperature applications. The CALPHAD methodology is a powerful tool for designing advanced lightweight materials by optimizing their compositions under specific heat-treatment conditions. Yet many mechanical properties (elastic constants, hardness, ductility, and yield strength), which are essential for the HEA design, cannot be directly obtained by this method. Recently, CALPHAD-based design has been integrated with theory-guided design and ML to overcome this problem. For example, Rao et al.^23^ developed a theory-guided design for lightweight, high-strength, and ductile single-phase refractory BCC HEAs. Using this method, they screened thousands of HfMoNbTaTi compositions to calculate their hardness, ductility, and yield strength, while thermodynamic data were obtained by CALPHAD. Likewise, Martin et al.^24^ developed a design method for lightweight HEAs using semi-empirical physicochemical rules^25,26^. The main disadvantage of this approach is its low prediction accuracy since the semi-empirical rules were based on simple classification algorithms, together with rather limited experimental data. Currently, the ML-based HEA design^19,27^ is considered as a viable alternative capable to overcome these difficulties by taking into account the complex non-linear relationships between the experimental and simulation data^17^.

All the methods considered above have advantages and disadvantages in terms of efficiency and accuracy. For example, although CALPHAD is an effective computational tool to explore phase stability, its methodology is based on databases for binary and ternary systems. Therefore, its predictions can be less accurate when the thermodynamic parameters are extrapolated to HEAs^9^. Importantly, some of the lightweight constituent elements, such as Mg, Li, Be, et al. are currently not included in the CALPHAD database^28^, hindering its application for the design of lightweight HEAs. Moreover, mechanical properties cannot be calculated by this method^9^. Due to these reasons, application of CALPHAD to HEA design, especially for lightweight HEAs, remains a challenge^16,19^. The hardness, ductility, and yield strength of HEAs can in principle be obtained by MD simulations, yet the lack of reliable interatomic potentials for lightweight HEAs limits its application. Moreover, it is a semi-empirical method, and its predictions often encounter significant uncertainty in terms of accuracy due to the empirical nature of interatomic potentials, along with the limitations on the length and time scales which may be beyond the reach of MD.

The main advantage of DFT-based calculations for HEA design is its versatility and computational accuracy in comparison with other methods. To our knowledge, four major first-principles-based methods have been developed to study HEAs: the first is the coherent potential approximation (CPA)^16,29–31^, the accuracy of which is limited by its mean-field nature where all the local effects are neglected^32^. The second is also a mean-field approach based on the virtual crystal approximation (VCA)^33–36^. The third is based on the special quasi random structures (SQS)^37–39^, which is more accurate, but its application to HEAs is hampered by its comparatively high computational cost.

The last method is based on the small set of ordered structures (SSOS)^16,40–44^, which is currently considered as the most promising approach for high-fidelity (HF) and high-throughput (HT) computations of HEAs. In the SSOS method, the properties of an HEA are calculated as a weighted average over a selected set of small ordered structures (SOS)^16,40^. The most important advantage of the SSOS method is a substantial reduction in the number of atoms required to model HEAs, since the SOS sample contains only a few atoms per unit cell. The method was initially proposed for equimolar HEAs^40^, and only a single SOS solution was identified. Subsequently, a set of SSOS solutions were found, and an averaging scheme was proposed to deal with their multiplicity^42^. The method was extended to handle non-equimolar compositions^43^ and to include short range order^44^. Recently, the modified preselected small set of ordered structures (PSSOS) method has been developed^41^, which significantly outperforms the original SSOS approach in terms of efficiency, making HF and HT screening of HEAs a reality.

In this work, we propose to use PSSOS for the design of lightweight HEAs in a single solid-solution (SS) phase. To design lightweight HEAs, one needs to consider light constituent elements like Li (${m}_{Li}=$ 6.9 amu), Be (${m}_{Be}=$ 9.1 amu), Mg (${m}_{Mg}=$ 24.3 amu), Al (${m}_{Al}=$ 26.9 amu) and Si (${m}_{Si}=$ 28.1 amu). Li and Be are selected because they reduce the density and increase the Young’s modulus of HEAs^45^. This synergetic property leads to a significant increase in the specific stiffness. Si is the most important alloying element, which is primarily responsible for good castability and low density. The addition of Mg markedly increases the strength of HEAs without overly decreasing the ductility^46^. Ti is added to strengthen HEAs and to increase their ability to withstand extremes of temperature^6^. Some of these lightweight elements, namely Be and Li, are typically not used in traditional HEAs since they tend to have a large atomic radius, electronegativity, and large difference in melting and boiling point^45^. Also, it can be difficult to experimentally handle some of those light elements, especially Be and Li, as they are considered to be hazardous materials^47^. In addition, computational design based on CALPHAD is not feasible because some of these lightweight elements are not included in the current CALPHAD database^21^. Equally, one cannot use MD simulations to study these HEAs with these lightweight elements since reliable interatomic potentials describing interaction between these lightweight elements are not available^48^.

At present, only the first principles-based calculations can be used to design HEAs that include these lightweight elements. Practically, PSSOS is the only method that allows the exploration of the entire compositional space of HEAs containing these lightweight elements. Our previous study^41^ has convincingly demonstrated that with a comparable accuracy, the PSSOS method is much faster than SSOS and SQS, making it feasible for HF, HT calculations and screening HEAs. In this work, we employ PSSOS to study the entire composition spaces (containing ~ 9000 compositions) of three quinary lightweight HEA families, that is, AlBeMgTiLi, AlBeMgTiSi and AlBeMgTiCu with both FCC and BCC lattice structures in the ideal solid-solution (SS) phase. It is noted that along with the lightweight elements, Be, Mg, Li, Si and Al, two comparatively light elements, that is, Ti (${m}_{Ti}=$ 47.9 amu) and Cu (${m}_{Cu}=$ 63.6 amu), are also included. Among these three families, four elements are the same, and only one element (Li, Si, or Cu) is different. In our study, we focus on the role of these elements, namely, we investigate how the inclusion of Li, Si, and Cu affects the physical properties (formation energy, mass density, and elastic constants) of the HEAs.

The following design criteria for the selection of best HEA compositions were considered: (1) high energetical stability, (2) lightweight, (3) large elastic modulus, (4) large specific stiffness, and (5) high Pugh’s ratio (which indicates the alloy ductility vs. its brittleness). The five topmost compositions were selected according to the design criteria for each of the three HEA families. The present work, for the first time, employed HT and HF first-principles-based methods to design lightweight HEAs.

It is widely acknowledged that designing and fabricating lightweight HEAs with stable structures and superior mechanical properties remains a challenge. Our study reveals that HEAs with the topmost compositions exhibit a negative formation energy, low mass density and high specific Young’s modulus, but a low Pugh’s ratio. These results indicate that the designed lightweight HEAs can be energetically more stable, lighter, and stiffer compared to existing Al alloys. However, they may exhibit a low ductility. These findings will provide valuable insights and guidance for experimental development and fabrication of lightweight HEAs with desirable properties.

## The computational method

### Outline of the SSOS method

**Figure 1:**
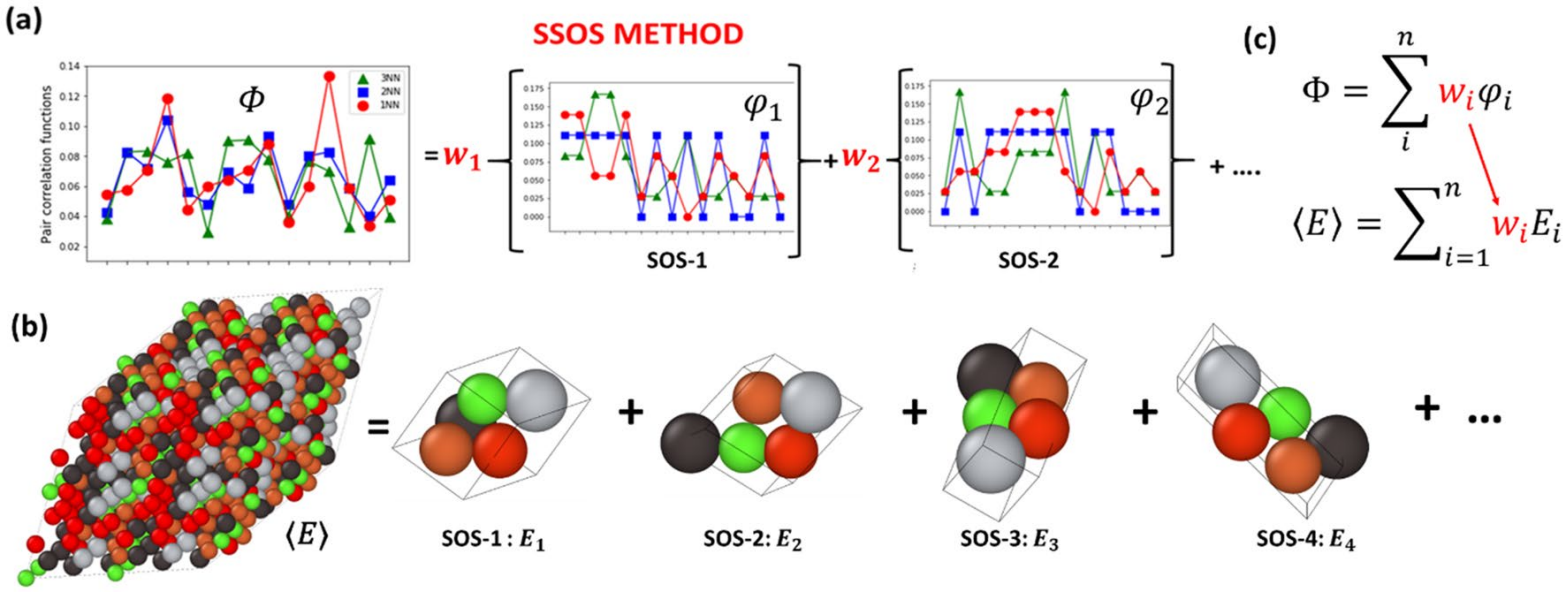
(**a**, **b**) Graphical representation of the SSOS method: (**a**) For a given composition of a quinary HEA in the ideal solid solution, one calculates the pair correlation functions up to 1st (red circles), 2nd (blue squares) and 3rd (green triangles) nearest neighbor range which are precisely matched by a linear combination of the weighted pair correlation functions of the selected SOS. The pair correlation functions are matched up to 3rd nearest neighbor range. (**b**) The target HEA sample and a set of 5-atom SOS with their pair correlation functions shown at the top panel (**c**). The HEA energy is calculated as a weighted average over the energies of the selected SOS, with the weights taken from the linear combination of weighted pair correlation functions.

The key idea of the SSOS method^16,32,40,42^ is to use a set of SOS to model an HEA with a given composition in the ideal solid-solution phase. The symmetry-unique SOS are constructed by using non-conventional, non-primitive unit cells of cubic lattices (BCC and FCC). The examples of SOS containing a small number of atoms per unit cell are shown in Fig. 1. At least 5 constituent atoms per unit cell are required for a quinary HEA. To model non-equimolar compositions, we extended the number of atoms to 6 and 7 per non-primitive unit cell^40,42^.

An SOS is uniquely characterized by its own pair correlation functions, {${\phi }_{i}\},$ describing the atomistic neighborhood of every constituent element within a specific range and serving as its distinctive identification (‘fingerprints’, see Fig. 3a). For each SOS, we calculated its atomic pair-correlation functions up to 3rd nearest neighbor (NN) range. In total, we found an extensive set of 5-, 6- and 7-atom SOS whose atomic pair-correlations can be used to model HEAs with non-equimolar compositions. For example, the complete set of all feasible SOS for a BCC lattice contains ${N}_{5}$ = 1,614 samples with 5-atoms per unit cell, ${N}_{6}$ = 14,560 with 6-atoms, and ${N}_{7}$ = 35,665 with 7-atoms per unit cell (in total ${N}_{bcc}=$ 51,839 SOS).

The complete set of all possible SOS is constructed first, then, a minimal-size subset of SOS is selected from it by matching the pair-correlation functions of a given HEA composition by a linear combination of the pair correlation functions of the selected minimal-size SOS subset. For the ideal SS phase, the target pair-correlation functions, ${\Phi }_{T},$ can be calculated analytically^44^. To find the SOS and the corresponding weights, we applied the stochastic hill climbing search method^49^ to minimize the deviation, $\lambda$, from the target pair correlation functions: $\lambda = \left( {{\Phi }_{T} - \sum\nolimits_{i}^{n} {w_{i} \phi_{i} } } \right)^{2} .$ A relatively large number ($\sim$ 100) of different SSOS solutions^40,42^ can be obtained when the target pair-correlation functions are matched up to 3^rd^ NN range.

The formation energy, mass density, and elastic moduli were calculated as a weighted average over the chosen SOS, which represents an SSOS solution (see Fig. 1) for a given HEA composition. We note that a set of different SSOS solutions can be found for a given composition. As one might expect the different SSOS solutions represent the same ideal solid solution state with a given compositions. In our approach, we did not differentiate between the diverse SSOS solutions, but averaged over them to obtain the energy and elastic moduli for a given HEA composition^42^. The energy and elastic moduli were calculated in two stages: first, a weighted average over SOS samples constituting a given SSOS solution was calculated, and then a simple average over the selected SSOS solutions was obtained. For example, to calculate the ground state energy (per atom), $$\left\langle {{\text{E}}_{{\text{g}}} } \right\rangle$$, of a given HEA composition, we first obtained the ground state energy (per atom)$$, {{\text{E}}}_{{\text{g}},{\text{s}}},$$ for each SSOS solution, s, according to:$${{\text{E}}}_{{\text{g}},{\text{s}}}=\sum_{{\text{i}}}^{{\text{n}}}{{\text{w}}}_{{\text{i}}}^{{\text{s}}}{{\text{E}}}_{{\text{gi}},{\text{s}}}^{{\text{sos}}}$$, where $${{\text{w}}}_{{\text{i}}}^{{\text{s}}}$$ is the weight coefficient of the ith SOS obtained for the $${\text{s}}$$-SSOS solution, $${{\text{E}}}_{{\text{gi}},{\text{s}}}^{{\text{sos}}}$$ is the energy (per atom) of the ith SOS of the $${\text{s}}$$-SSOS solution, and n is the number of SOS samples. The ground state $${{\text{E}}}_{{\text{gi}},{\text{s}}}^{{\text{sos}}}$$ for a given SOS structure was obtained by DFT calculations when the preselected set was DFT optimized. Next, the $$<{{\text{E}}}_{{\text{g}}}>$$ was obtained as an average over the set of SSOS solutions $$<{{\text{E}}}_{{\text{g}}}>= \frac{1}{{\text{m}}} \sum_{{\text{s}}}^{{\text{m}}}{{\text{E}}}_{{\text{g}},{\text{s}}}$$, where $${\text{m}}$$ is the number of SSOS solutions used for the averaging procedure ($${\text{m}}$$=5 in our case). The obtained $$<{{\text{E}}}_{{\text{g}}}>$$ was then used to calculate the formation energy per atom for an ABCDE HEA with a given composition $$[c\left(A\right),c\left(B\right),c\left(C\right),c\left(D\right),c\left(E\right)].$$ The formation energy was obtained by subtracting the energy of constituent atoms, $${{\text{E}}}_{{\text{ROM}}}$$ energy (per atom$$),$$ from the $${<{\text{E}}}_{{\text{g}}}>$$. The $${{\text{E}}}_{{\text{ROM}}}$$ was calculated by the rule of mixtures: $${{\text{E}}}_{{\text{ROM}}}={\text{c}}\left({\text{A}}\right){{\text{E}}}_{{\text{A}}}+{\text{c}}\left({\text{B}}\right){{\text{E}}}_{{\text{B}}}+{\text{c}}\left({\text{C}}\right){{\text{E}}}_{{\text{C}}}+{\text{c}}\left({\text{D}}\right){{\text{E}}}_{{\text{D}}}+{\text{c}}\left({\text{E}}\right){{\text{E}}}_{{\text{E}}}$$, where, $${{\text{E}}}_{{\text{A}}}$$ is the ground state energy per atom of $${\text{A}}$$-atom in its pure most energetically stable crystalline phase^42^, $${{\text{E}}}_{{\text{B}}}$$ is the ground state energy per atom of $${\text{B}}$$-atom in its pure most energetically stable crystalline phase, and so forth. The elastic moduli were calculated in the same way.

The SOS solutions were taken from a relatively small preselected, DFT pre-optimized set with ~ 2000 SOS. When optimizing the entire preselected set, we calculated with DFT the ground state energy and elastic moduli for each SOS structure, therefore around ~ 2000 DFT runs were performed for the entire compositional space of a selected lightweight HEA. Although this number of DFT simulations seems to be large, we notice that each SOS structure is a generally non-primitive, non-orthogonal unit cell for cubic lattice containing between 5 to 7 constituent atoms. This small sized samples can be efficiently optimized by DFT, especially by using VASP^50^.

To deal with the non-uniqueness of SSOS solutions, we calculated the physical properties as an average over the obtained solutions^42^. The HEA properties were calculated in two steps: first, a weighted average over SOS samples constituting a given SSOS solution was calculated, and then a simple average over several SSOS solutions was obtained. For example, the ground state energy per atom of a given HEA, $<{E}_{g}>$, is calculated in the following way: First, the ground state energy of per atom $, {E}_{g,s},$ is obtained for each SSOS solution, *s*:${E}_{g,s}=\sum_{i}^{n}{w}_{i}^{s}{E}_{gi,s}^{sos}$, where ${w}_{i}^{s}$ is the weight coefficient of the *i*th SOS obtained for the $s$-SSOS solution, ${E}_{gi,s}^{sos}$ is the ground state energy per atom of the *i*th SOS of the $s$-SSOS solution, and *n* is the number of SOS samples. Next, the $<{E}_{g}>$ is obtained as $<{E}_{g}> = \frac{1}{m} \sum_{s}^{m}{E}_{g,s}$, where $m$ is the number of SSOS solutions used for the averaging procedure ($m$ = 5 in our case). The other physical properties are calculated in the same manner. To calculate the formation energy per atom for an ABCDE HEA with a given composition [$c\left(A\right),c\left(B\right),c\left(C\right),c\left(D\right),c\left(E\right)$], its ground state energy per atom, $\left\langle {E_{g} } \right\rangle$*,* was obtained first. After that the ${E}_{ROM}$ energy per atom was subtracted from the $\left\langle {E_{g} } \right\rangle$*.* The ${E}_{ROM}$ is calculated by the rule of mixtures: ${E}_{ROM}=c\left(A\right){E}_{A}+c\left(B\right){E}_{B}+c\left(C\right){E}_{C}+c\left(D\right){E}_{D}+c\left(E\right){E}_{E}$, where, ${E}_{A}$ is the ground state energies per atom of $A$-atom in its pure most energetically stable crystalline phase^42^.

### Outline of the PSSOS approach

Although significant progress has been made in developing the SSOS method, a few remaining challenges limit its application for exploring the entire composition space of HEAs. First, the total number of SOS structures used to construct SSOS solutions for the entire design space of HEAs is huge, causing difficulty in selecting a minimal set for SSOS solutions. Second, the DFT calculations for so many SOS structures are too expensive and impractical, making the SSOS method lose its computational efficiency. To overcome these problems, we adopted a new approach based on the PSSOS method. The PSSOS method is an improvement of the original SSOS method^41^. In the original SSOS method, all the SOS structures required by PSSOS method were generated by Alloy Theoretic Automated Toolkit (ATAT)^38^. However, the total number of SOS structures required to construct SSOS solutions for the entire design space of HEAs is enormous, making the selection of a minimal set for SSOS solutions a challenge. Besides that, the DFT optimization of all the feasible SOS structures is too expensive, making the SSOS method lose its computational advantage over the SQS-based method. The new PSSOS technique gets around these problems by constructing a small, preselected set of SOS.

First, we identified all feasible SSOS extended solutions (infra vide) by screening the entire compositional space. Then the SOS structures were selected in direct proportion to their frequency of appearing in the SSOS solutions. The number of SOS structures in the preselected set is a relatively small set of ~ 2000 SOS in comparison to the complete set of all feasible structures, ~ 52,000 SOS, so the whole preselected set can be efficiently optimized by DFT.

In the original SSOS approach, one needs to search for a minimal set of SOS (containing 5, 6 or 7 atoms) in an SSOS solution, selected from the complete set of all feasible SOS, and subsequently optimizes their geometries by DFT. The search for the minimal set is an extremely challenging problem in the enormous complete SOS set with ~ 52,000 SOS, and the number of identified SOS can be large. On the contrary, in the PSSOS approach, we extend the SSOS solution set beyond the minimal size. The extended set solutions can be easily obtained by comparison with the minimal set solutions. Moreover, the complete SOS set is not required to find the extended solutions, since the required number of SOS for the extended solution can be chosen from a relatively small preselected, DFT preoptimized set with ~ 2000 SOS. Since all the SOS in the obtained SSOS solution are taken from the same preselected set, the computational cost of the set expansion is negligible. We demonstrated that with comparable accuracy, the PSSOS method is much faster than SSOS and SQS, making it feasible for HF, HT calculations and screening HEAs^41^.

In application of the SSOS method, we use a small set of ordered structures containing 5–7 atoms. The selected SOS structures are ordered, infinitely large crystalline lattices (periodic boundary conditions are applied in all three directions). Therefore, if the number of atoms in an SOS structure is increased by the sample replication along the three directions, the corresponding physical properties obtained by DFT calculations will be identical to those of the original small atom structure. Hence, the size scaling of SOS samples does not affect the obtained results.

### Systematic exploration of the composition space

**Figure 2:**
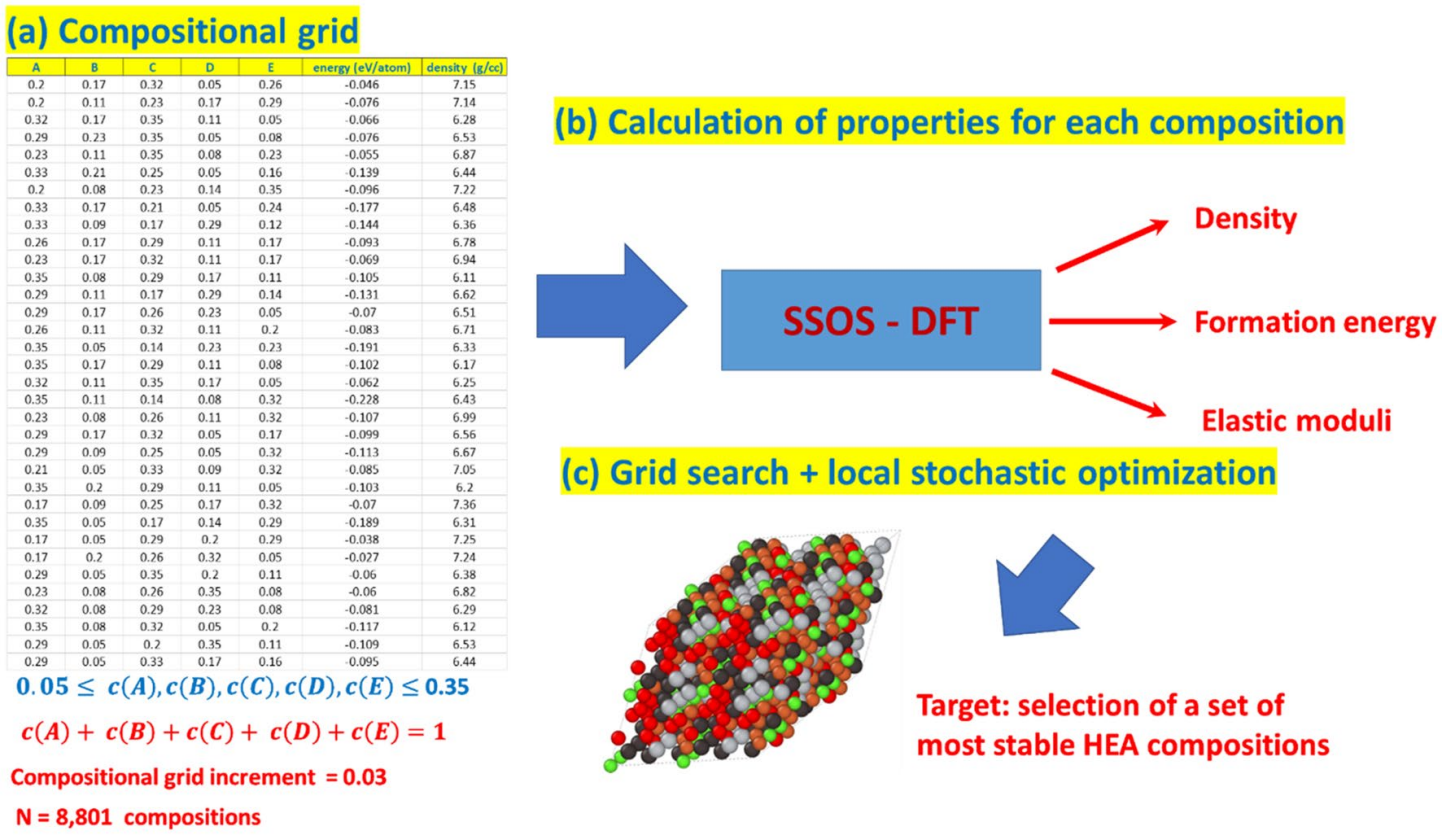
Schematic representation of the HEA design by the PSSOS method: (**a**) A grid representing of the composition space with an incremental grid step of ∆ = 3% contains 8,801 HEA compositions. The constraints on the molar fractions of constituent elements are indicated. (**b**) Calculation of the materials properties such as formation energy per atom, mass density and elastic moduli for all the constructed HEA compositions with BCC and FCC lattices. (**c**) Selection of the topmost HEA compositions and the consequent off-grid search for the best possible solutions.

A composition grid with sufficiently accurate resolution (the small increment step in the compositional space) is prerequisite for the comprehensive examination of the entire compositional space of a given lightweight HEA, where we are looking for the topmost compositions with the best physical properties, for example, the mass lowest density and the highest specific Young’s modulus. By selecting the 3% increment in the compositional space, we ensure the sufficient accuracy in the examination of the compositional space. This results in 8801 selected compositions in total: one equimolar and 8800 non-equimolar compositions (see Fig. 2). For each selected grid composition, we identified a set of SOS structures (SSOS solution), which, with the appropriate weighting coefficients, can describe the physical properties of the selected composition. If one is seeking for a minimal size SSOS solution, i.e., a solution containing the minimum number of SOS structures, it can be obtained by searching through the entire set of all the possible 5-, 6- and 7-atom SOS with BCC (or FCC) lattice. The entire set of SOS is vast since it contains ~ 52,000 samples. However, if one is looking for a moderately small size SSOS solution, instead of a minimal size SSOS solution, we found that a relatively small subset of the entire set containing about ~ 2000 SOS structures is sufficient: this number is independent of the number of selected grid compositions. We call this relatively small subset of DFT-optimized SOS the preselected small set of ordered structures (PSSOS).

To construct the composition grid, we set the lower (5%) and upper (35%) boundaries for the molar fraction of constituent elements and set the grid increment in molar fraction as $\Delta =$ 3%. Although a quinary HEA contains five constituent elements, only four out of five molar fractions of the constituent elements are linearly independent since the sum of them is equal to one: $c\left(A\right)+c\left(B\right)+c\left(C\right)+c\left(D\right)+c\left(E\right)=1.$ Therefore, to construct the composition grid of ABCDE HEA, we systematically varied the molar fraction of $A$, $B$, $C$ and $D$ within the specified range, while the molar fraction of $E$ was obtained as a remainder. We generated the selected grid increment (see Fig. 2) and identified the topmost compositions according to the specific design criteria. Next, we chose five out of the topmost compositions and used the stochastic hill climbing method^49^ to examine their off-grid neighborhood in search of the optimized solutions.

The original SSOS method was carefully validated in^40^. In one of our previous papers [“A first‑principles‑based high fidelity, high throughput approach for the design of high entropy alloys” published in^41^, we have validated our extension of the original SSOS approach to the PSSOS method by comparing our predictions with the data from already established experimental and computational studies. The comparisons demonstrate that the results obtained from the present study are in good agreement with the findings reported previously^11,12,15,44^, validating our computational method.

To the best of our knowledge, we are unaware of any experimental results for the selected lightweight alloys (AlBeMgTiLi, AlBeMgTiSi, and AlBeMgTiCu) that can be used for the direct comparison with our results. Yet, our predictions for the low density and high brittleness of the lightweight HEAs are in line with the available experimental data for similar lightweight HEAs^7^.

Ideally, a theory should serve as a compass to guide the experimental research rather than merely trailing it. In alignment with this philosophy, we have conducted an extensive computational search for the topmost lightweight HEA with the required mechanical properties. While we acknowledge the significance of experimental data, our current work focuses on computational analyses to shed light on the design guideline for lightweight HEAs.

### Details of the DFT calculations

Our DFT calculations were carried out with the generalized Perdew-Burke-Ernzerhof^51^ and the projector-augmented wave (PAW) pseudopotential plane-wave method^52^, as implemented in the VASP code^50^. We calculated the elastic constants by deforming SOS samples and deriving their elastic constants from the strain–stress relation. The corresponding elastic moduli (Young’s, bulk and shear modulus) were obtained from the elastic constants by using the Voigt approximation scheme^53^. In our DFT calculations, we used 12 × 12 × 12 Monkhorst-Pack^54^ k-point grid for optimization of the unit cell geometry and calculation of the formation energy. A plane-wave basis set with an energy cut-off of 520 eV was adopted. Good convergence was obtained with these parameters. For example, the total energy converged to 10^−7^ eV per atom. Spin polarized calculations were implemented in our study. Energy minimization was accomplished by using the conjugate-gradient optimization to relax the atomic positions without constraining lattice constants.

## Results

We calculate the physical properties for the chosen lightweight HEAs with BCC and FCC lattices: AlBeMgTiSi, AlBeMgTiCu, and AlBeMgTiLi. We found that for these HEAs the formation energies per atom of the topmost energetically stable compositions with BCC lattice are lower than those with FCC. Therefore, we focus only on the HEAs with BCC lattice in a single SS phase. We also identified the most stable compositions with the lowest mass density and the highest elastic constants, specific stiffness and Pugh’s ratio by systematically screening each HEA system’s entire compositional space.

### AlBeMgTiSi

#### Formation energy

**Figure 3:**
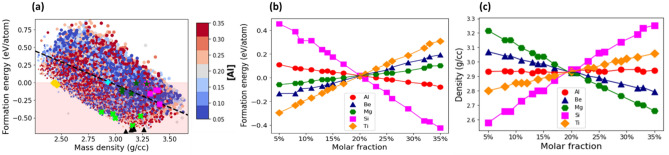
(**a**) The formation energy per atom versus the mass density for the grid compositions of AlBeMgTiSi with BCC lattice in single SS phase. The marker color indicates the molar fraction of Al as shown by the side colorbar and the marker size is proportional to the molar fraction of Ti. The shaded region marks only the energetically stable compositions. The topmost stable compositions with the lowest energy indicated by black triangles, with the lowest mass density by yellow diamonds, with the highest elastic modulus by magenta squares, with the largest Pugh’s ratio by green crosses, and with the highest specific elastic modulus by green stars. The equimolar composition is specified by cyan plus marker. The dashed line indicates correlation between the formation energy and mass density. (**b**, **c**) The mean formation energy (**b**) and mass density (**c**) as a function of molar fraction of its constituent elements. The mean values are calculated as an average over compositions with a given molar fraction of a constituent element.

Figure 3a shows the formation energy per atom versus the mass density for all the grid compositions of AlBeMgTiSi. The stable compositions are located within the shaded region. As can be seen in Fig. 3a, the data points form a quadrilateral pattern, which is characterized by the broad distribution of the data points along the dashed line with a negative slope. The slope describes a negative correlation between the formation energy and mass density: the higher the composition density, the lower the formation energy.

The topmost energetically stable AlBeMgTiSi compositions (black triangles in Fig. 3a) are in the density range between ρ = 3.0 g/cm^3^ and ρ = 3.3 g/cm^3^. The topmost stable compositions (yellow diamonds) with the lowest density are in the range between ρ = 2.4 g/cm^3^ and ρ = 2.6 g/cm^3^. They do not overlap with the topmost energetically stable compositions. The topmost stable compositions with the largest Young’s modulus (magenta squares) and specific stiffness (green stars) are scattered in the range between ρ = 3.3 g/cm^3^ and ρ = 3.6 g/cm^3^. Likewise, the topmost stable compositions with the highest Pugh’s ratio (green crosses) are predominantly in the low-density range. As can be seen in Fig. 3a, the most stable compositions with the lowest formation energy, the composition with the lowest mass density, as well as the compositions with the highest elastic modulus, specific stiffness and Pugh’s ratio occupy separated regions in the compositional space. Therefore, it is challenging to find the compositions with the lowest formation energy and density, along with the highest elastic modulus, specific stiffness, and Pugh’s ratio. Hence, the specific physical properties of AlBeMgTiSi can be optimized with some tradeoffs in others.

Next, we investigated the effect of constituent elements on the formation energy. The highly energetically stable compositions of AlBeMgTiSi are in the vicinity of the most energetically stable ones (black triangles in Fig. 3a). The molar fraction of Al is indicated by the marker color Fig. 3a. The marker color of highly stable compositions varies from red to blue: One can identify the compositions with both high and low molar fractions of Al. To illustrate the effect of the molar fraction of Al, we calculated the mean formation energy as an average over all the compositions with a given molar fraction of Al (Fig. 3b). The relatively small slopes of the mean formation energy for Al (red circles), as well as for Be (blue triangles) and Mg (green hexagons) indicate that the mean formation energy depends weakly on the variation in their molar fractions. By contrast, the large negative slope for Si (magenta squares) suggests that the formation energy is greatly affected by its molar fraction. The stability of AlBeMgTiSi is significantly improved at high molar fraction of Si. The large positive slope for Ti (yellow diamonds) indicates that it reduces the stability of AlBeMgTiSi, especially at high molar fractions (of Ti).

We note that the mean formation energy, as defined above, serves only to indicate the average trend, and should not be overinterpreted. A better description of the effect of molar fraction of a constituent element on the formation energy (and the mass density) is provided in Fig. 3a and in Supplementary Fig. S1, where the marker color indicates the molar fraction of a selected constituent element in each composition. For example, in Supplementary Fig. S1a, the marker color indicates the molar fraction of Be. The marker color distribution in the domain of highly energetically stable compositions (see the region adjacent to black triangles in Supplementary Fig. S1a) is mixed: One can locate the energetically stable compositions with both high and low molar fractions of Be. Likewise, the red and blue colored markers corresponding to the compositions with both high and low molar fractions of Mg can be found in the same area (see Supplementary Fig. S1b). By contrast, all the highly stable compositions contain only the highest molar fraction of Si (red markers near black triangles in Supplementary Fig. S1c) or the lowest molar fraction of Ti (blue markers in the same area in Supplementary Fig. S1d). We found that the most energetically stable composition of AlBeMgTiSi (Table 1) contains the highest molar fraction of Si (35%) and the lowest of Ti (5%). The five topmost stable compositions of AlBeMgTiSi are listed in Supplementary Table S1.

**Table 1:** The most energetically stable compositions of the selected HEAs sorted in ascending order according to their values.

HEA	Composition	Formation energy (eV/atom)	Free energy at T = 300K (eV/atom)	GCLP free energy (eV/atom)	Mass density (g/cc)	Young’s modulus (GPa)	Specific Young’s modulus (MN/kg)	Pugh’s ratio (B/G)
AlBeMgTiSi	[0.31, 0.15, 0.14, 0.05, 0.35]	0.691	− 0.803	− 0.152	2.43	135.77	55.91	0.95
AlBeMgTiCu	[0.32, 0.05, 0.05, 0.35, 0.23]	− 0.155	− 0.187	− 0.351	4.58	166.73	36.40	1.33
AlBeMgTiLi	[0.35, 0.05, 0.14, 0.32, 0.14]	− 0.074	− 0.111	− 0.326	3.01	156.4	52.05	1.28

The mass density, elastic modulus, specific stiffness, and Pugh’s ration are reported for each composition.

#### Mass density

The mean mass density of AlBeMgTiSi as a function of the molar fraction of its constituent elements is shown in Fig. 3c. We calculated the mean mass density as an average over all the compositions with a given molar fraction of a specific constituent element. The mean mass density decreases linearly with increasing molar fraction of Be (blue triangles) and Mg (green hexagons). The negative slope for Be is the largest since this lightest element greatly reduces the mass density of AlBeMgTiSi. In Supplementary Fig. S1a, where the marker color indicates the molar fraction of Be, the compositions with the lowest density (see the area near yellow diamonds) contain the highest molar fraction of Be: The color of the markers is predominantly red. Similarly, the color of the markers is exclusively red for Mg in Fig. S1b. In contrast to this, the positive slope of the mean mass density for Si (magenta squares) and Ti (yellow diamonds) indicates that the mass density increases with increasing the molar fraction of these elements. The effect is particularly strong for Ti, which is the heaviest constituent element in AlBeMgTiSi. In Supplementary Fig. S1d, where the marker color indicates the molar fraction of Ti, the compositions with the lowest mass density contain the lowest molar fraction of Ti: The color of the markers is entirely dark blue. In a similar fashion, the color of the markers is blue for Si in Supplementary Fig. S1c.

The slope for Al is negligibly small, suggesting that the mass density of AlBeMgTiSi is almost unaffected by the variation in its molar fraction. As can be seen in Fig. 3a, where the marker color indicates the molar fraction of Al, the compositions with the lowest mass density (the region near yellow diamonds) may contain both high and low molar fractions of Al. A combination of blue and red colored markers represents these compositions. We found that the lightest stable composition of AlBeMgTiSi (Table 2) contains the highest molar fraction of Be and Mg (35%) and the lowest of Ti $($5%). The five topmost stable compositions of AlBeMgTiSi with the lowest mass densities are reported in Supplementary Table S2.

**Table 2:** The most lightweight stable compositions listed in an ascending order according to their values.

HEA	Composition	Mass density (g/cc)	Formation energy (eV/atom)	Young’s modulus (GPa)	Specific stiffness (MN/kg)	Pugh’s ratio (B/G)
AlBeMgTiLi	[0.35, 0.05, 0.14, 0.11, 0.35]	2.07	− 0.004	88.88	43.02	1.26
AlBeMgTiSi	[0.11, 0.35, 0.35, 0.05, 0.14]	2.43	− 0.003	135.77	55.90	0.95
AlBeMgTiCu	[0.35, 0.08, 0.29, 0.23, 0.05]	3.03	− 0.004	144.57	47.65	1.30

In addition, we report the formation energy, elastic modulus, specific stiffness, and Pugh’s ratio for each composition.

#### Elastic modulus

**Figure 4:**
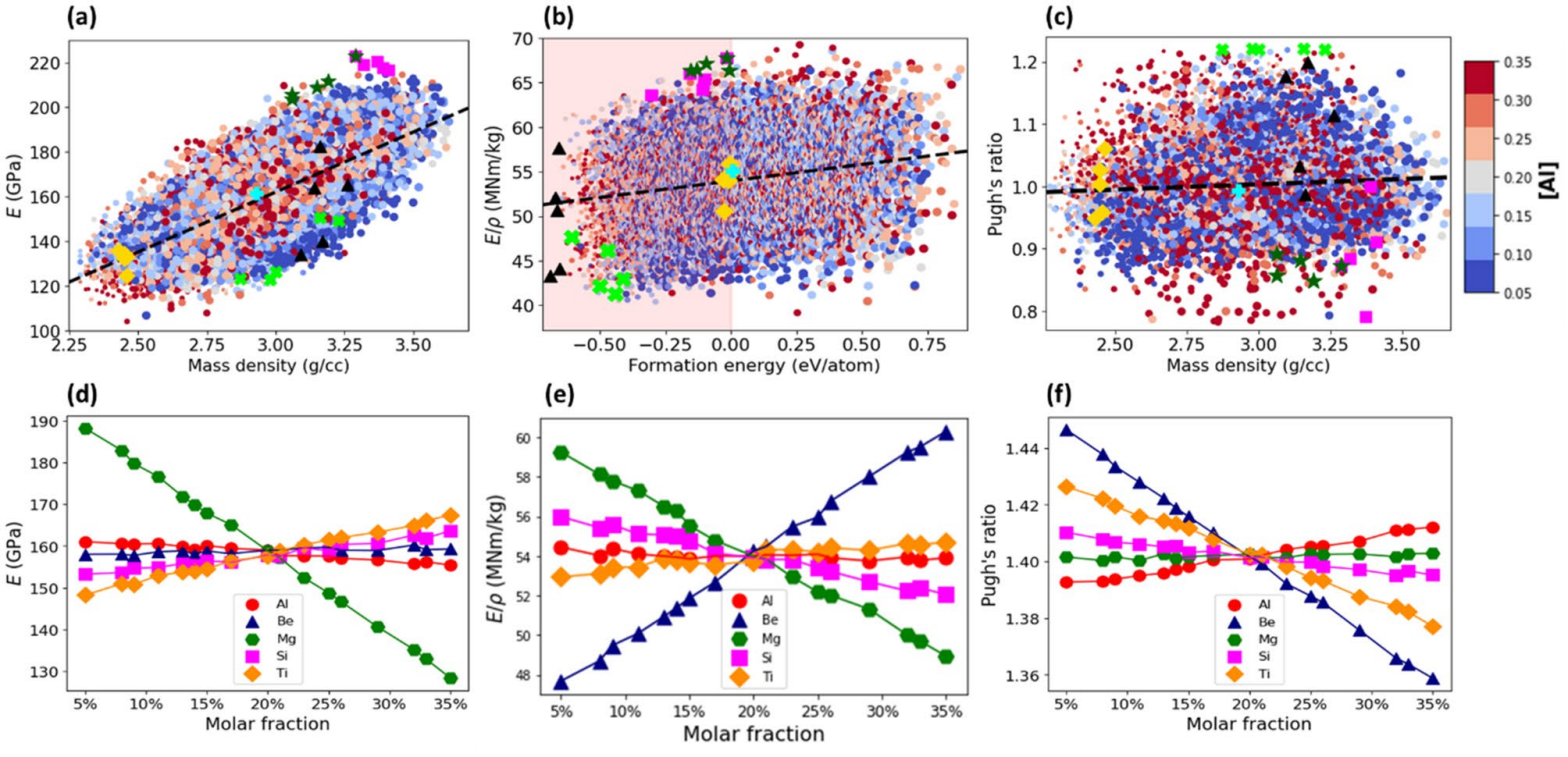
(**a**) The Young’s modulus versus the mass density for the grid compositions of AlBeMgTiSi with BCC lattice in single SS phase. (**b**) The specific stiffness versus the formation energy. The shaded region contains only the stable compositions. (**c**) The Pugh’s ratio versus the mass density. The color of the markers indicates the molar fraction of Al as shown by the side colorbar and the size of the markers is proportional to the molar fraction of Ti. The dashed line indicates correlation between the elastic modulus and density in (**a**), specific stiffness and formation energy in (**b**), and Pugh’s ratio and density in (**c**). The topmost stable compositions with the lowest energy indicated by black triangles, with the lowest mass density by yellow diamonds, with the highest elastic modulus by magenta squares, with the highest stiffness by green stars, and with the largest Pugh’s ratio by green crosses. The equimolar composition is specified by cyan plus marker. The mean elastic modulus (**d**), specific stiffness (**e**) and Pugh’s ratio (**f**) as a function of molar fraction of its constituent elements.

Figure 4a plots the elastic modulus, $E$, versus the mass density, *ρ,* for all the grid compositions of AlBeMgTiSi. The data points form an elliptically shaped pattern. The major axis of the pattern is oriented along the dashed line. The positive slope of the line indicates a positive linear correlation between *E* and *ρ*: the higher the density, the larger the elastic modulus.

The mean elastic modulus of AlBeMgTiSi as a function of the molar fraction of its constituent elements is shown in Fig. 4d. It is calculated in the same way as the mean formation energy or density. As can be seen in Fig. 4d, the higher the molar fraction of Si (magenta squares), Ti (yellow diamonds), and Be (blue triangles), the larger the elastic modulus. In comparison to Si and Ti, the effect of the molar fraction of Be on *E* is relatively weaker. In Supplementary Fig. S2a, where marker color indicates the molar fraction of Si, the compositions with the largest elastic modulus (see the region near the magenta squares) have the highest molar fraction of Si: The color of the markers is red. In a similar way, the color of the markers is predominantly red for Ti in supplementary Fig. S2b (and for Be in supplementary Fig. S2c). The marginally small slope for Al (red circles in Fig. 4d) implies the weak effect of Al on the elastic modulus. The molar fraction of Al is indicated by the marker color in Fig. 4a. The marker colors for the composition with high elastic modulus are mixed: One can find compositions with any value of the molar fraction of Al. By contrast, the large negative slope for Mg (green hexagons in Fig. 4d) indicates that an increase in its molar fraction reduces the elastic modulus. In Supplementary Fig. S2d, where the marker color indicates the molar fraction of Mg, the compositions with the largest elastic modulus contain low molar fractions of Mg: The marker colors are blue. The most stable composition with the highest elastic modulus (Table 3) contains the highest molar fraction of Si (29%) and the lowest molar fraction of Mg (5%). The topmost five stable compositions of AlBeMgTiSi with the highest elastic moduli are listed in Supplementary Table S3.

**Table 3:** The stable compositions with the largest Young’s modulus sorted in a descending order according to their values.

HEA	Composition	Young’s modulus (GPa)	Formation energy (eV/atom)	Mass density (g/cc)	Specific Young’s modulus (MN/kg)	Pugh’s ratio (B/G)
AlBeMgTiSi	[0.26, 0.15, 0.05, 0.25, 0.29]	222.85	− 0.017	3.29	67.78	0.87
AlBeMgTiCu	[0.25, 0.32, 0.05, 0.32, 0.05]	202.93	− 0.076	3.42	59.40	1.19
AlBeMgTiLi	[0.26, 0.32, 0.05, 0.32, 0.05]	193.45	− 0.027	3.12	62.05	1.21

For each composition we report the formation energy, mass density, specific stiffness, and Pugh’s ratio.

#### Specific stiffness

Figure 4b plots the specific stiffness, $E/\rho$, against the formation energy for all the grid compositions of AlBeMgTiSi. The shape of the specific stiffness versus formation energy pattern is characterized by the wide distribution of the data points. As can be seen in Fig. 4b, the topmost energetically stable compositions (black triangles), the lightest stable compositions (yellow diamonds) and the compositions with the highest Pugh’s ratio (green crosses) do not overlap. However, the compositions with the highest *E* (magenta squares) partially overlap with those with the highest $E/\rho$ (green starts).

We calculated the mean $E/\rho$ as an average over all the compositions with a given molar fraction of a given constituent element (Fig. 4e), in much the same manner as the mean formation energy. We found that an increase in the molar fraction of Be (blue triangles) can significantly increase $E/\rho$, while an increase in the molar fraction of Mg (green hexagons) may considerably reduce $E/\rho$. In Supplementary Fig. S3a, where the marker color indicates the molar fraction of Be, the compositions with the largest specific stiffness contain the highest molar fraction of Be: The color of the markers is dark red. In Fig. S3b, where the marker color indicates the molar fraction of Mg, the compositions with the largest specific stiffness are blue colored. For Al (Fig. 4b), Si (Supplementary Fig. 4c) and Ti (Supplementary Fig. 4d), the marker color of the composition with the highest $E/\rho$ is a mixture of red and blue: One can find the compositions with any molar fraction of these constituent elements. The top stable composition of AlBeMgTiSi with the largest specific stiffness (Table 4) contains the highest molar fraction of Be (35%) and the lowest fraction of Mg (5%). The five topmost stable compositions with the highest E*/ρ* are listed in Supplementary Table S4.

**Table 4:** The stable compositions with the highest specific stiffness sorted in a descending order according to their values.

HEA	Composition	Specific stiffness (MN/kg)	Formation energy (eV/atom)	Mass density (g/cc)	Young’s modulus (GPa)	Pugh’s ratio (B/G)
AlBeMgTiSi	[0.05, 0.35, 0.05, 0.3, 0.25]	67.78	− 0.017	3.29	222.85	0.87
AlBeMgTiLi	[0.33, 0.33, 0.05, 0.24, 0.05]	65.53	− 0.012	2.93	192.06	1.18
AlBeMgTiCu	[0.32, 0.35, 0.08, 0.2, 0.05]	60.71	− 0.006	3.19	193.96	1.17

For each composition, we report the formation energy, mass density, elastic modulus, and Pugh’s ratio.

#### Pugh’s ratio

According to Pugh^55^, the ductile–brittle transition is related to the ratio between bulk modulus, $B$, and shear modulus, $G$. Pugh found that $B/G$ correlates with the ductility of elemental metals, where $B$ is used to estimate the resistance to fracture, while $G$ the propensity for increased fracture resistance after the onset of plastic deformation. The Pugh’s ratio can be used as a phenomenological criterion for ductility. Figure 4c plots the distribution of the Pugh’s ratio ($B/G$) against the mass density for all the compositions of AlBeMgTiSi. The critical value of the Pugh’s ratio $B/G$ is 1.75: alloys with $B/G > 1.75$ are ductile, whereas those with $B/G < 1.75$ are brittle^55^. Although this can be an accurate quantitative estimate for pure metals and simple compounds, the ductility of HEAs is more intricate, and such predictions must be seen as qualitative. As can be seen from Fig. 4c, all the AlBeMgTiSi compositions according to the Pugh’s criterion are brittle. The marker color indicates the molar fraction of Al in Fig. 4c. Since the color of the markers for the compositions with the high Pugh’s ratio (see the area around green crosses) is red, they contain the high molar fraction of Al (see also Supplementary Fig. S4 for the effect of the molar fraction of the remaining elements). It can be seen in Supplementary Fig. S4a,b, the smaller the molar fraction of Be and Si (constituent elements taken from very brittle materials), the larger the Pugh’s ratio.

Figure 4f plots the mean value of Pugh’s ratio for AlBeMgTiSi as a function of the molar fraction of its constituent elements, which was calculated in much the same fashion as the mean formation energy. Evidently, an increase in the molar fraction of Al enhances the $B/G$, while the effect of Mg, Si, Ti, and especially Be is opposite. We found that the most stable AlBeMgTiSi composition with the highest Pugh’s (Table 5) contains the largest molar fraction of Al (28%) and the smallest of Be (11%). The five topmost stable compositions of AlBeMgTiSi with the highest Pugh’s ratio are listed in Supplementary Table S5.

**Table 5:** The stable compositions with the highest Pugh’s ratio sorted in a descending order according to their values.

HEA	Composition	Pugh’s ratio (B/G)	Formation energy (eV/atom)	Mass density (g/cc)	Young’s modulus (GPa)	Specific stiffness (MN/kg)
AlBeMgTiLi	[0.32, 0.05, 0.17, 0.18, 0.28]	1.56	− 0.002	2.53	98.82	38.99
AlBeMgTiCu	[0.13, 0.06, 0.14, 0.32, 0.35]	1.39	− 0.003	5.01	141.74	28.29
AlBeMgTiSi	[0.28, 0.11, 0.24, 0.15, 0.22]	1.22	− 0.031	2.46	132.94	54.13

For each composition, we report the formation energy, mass density, elastic modulus, and specific stiffness.

### AlBeMgTiLi

#### Formation energy

**Figure 5:**
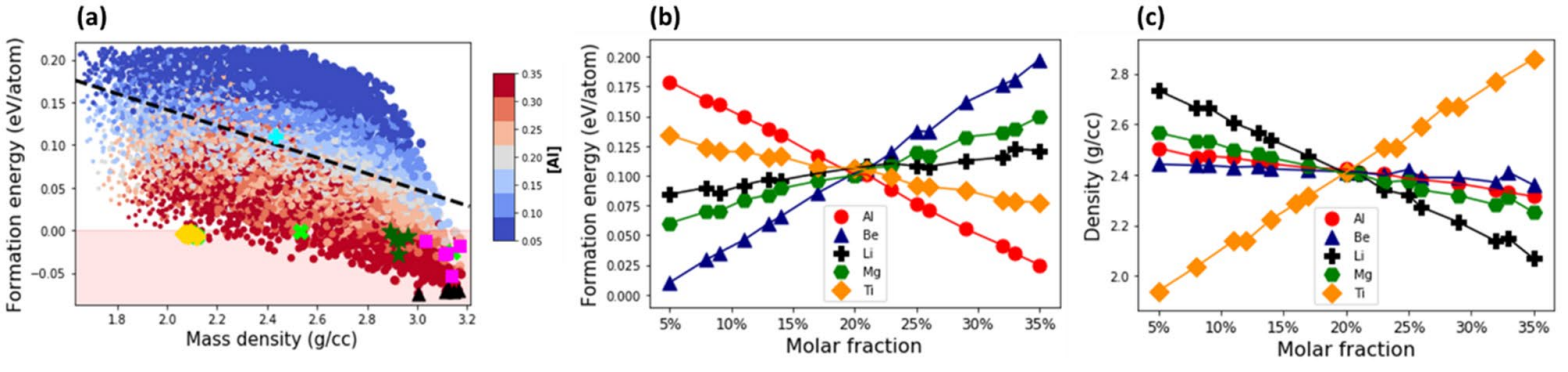
(**a**) The formation energy per atom versus mass density for all grid compositions of AlBeMgTiLi with a Body-Centered Cubic (BCC) lattice in a single Solid Solution (SS) phase. The color of the markers indicates the molar fraction of Al as shown by the side colorbar and the marker size is proportional to the molar fraction of Ti. The shaded region includes only the energetically stable compositions. The topmost stable compositions with the lowest energy indicated by black triangles, with the lowest mass density by yellow diamonds, with the highest elastic modulus by magenta squares, with the largest Pugh’s ratio by green crosses, and with the highest specific stiffness by green stars. The equimolar composition is specified by cyan plus marker. The dotted line indicates correlation between the formation energy and density. (**b**, **c**) The mean formation energy (**b**) and mass density (**c**) as a function of molar fraction of its constituent elements. The mean values are calculated as an average over compositions with a given molar fraction of a constituent element.

Figure 5a plots the formation energy versus the mass density for all the examined compositions of AlBeMgTiLi in single SS phase. In comparison to AlBeMgTiSi, the fraction of energetically stable compositions of AlBeMgTiLi is visibly smaller (${\nu }_{s}$ $\cong$ 20%). The data points scattered along the dashed line with a negative slope indicate that the compositions with higher mass density are more energetically stable.

The densities of the topmost energetically stable compositions are in the range between ρ = 3 g/cm^3^ and ρ = 3.2 g/cm^3^ (black triangles in Fig. 5a). The topmost stable compositions with the largest elastic modulus (magenta squares) and specific stiffness (green stars) are located close to this density region, but their formation energies are higher. The topmost stable compositions with the lowest density are in the density region between ρ = 2.1 g/cm^3^ and ρ = 2.2 g/cm^3^ (yellow diamonds). They overlap with several of the stable topmost compositions with the highest Pugh’s ratio (green crosses). Therefore, one can identify a few of the topmost compositions with the lowest mass density and the highest Pugh’s ratio.

The region with the highly stable compositions of AlBeMgTiLi is in the vicinity of the topmost energetically stable compositions (black triangles in Fig. 5a). It contains only the dark red colored markers, indicating the highest molar fraction of Al. Likewise, the relatively large negative slope of the mean formation energy as a function of the molar fraction of Al (red circles in Fig. 5b) implies the strong effect of Al: the higher its molar fraction, the lower the formation energy for the given composition. Similarly, the negative slope of the mean formation energy as a function of molar fraction of Ti (yellow diamonds) implies that Ti can stabilize AlBeMgTiLi, although its effect is comparatively weaker than that of Al.

As shown in Supplementary Fig. S5a, the region with the highly stable compositions contains only the dark red colored markers, indicating the highest molar fraction of Ti. The effect of Be, Li and Mg is the opposite to that of Al and Ti, as indicated by their positive slopes of the mean formation energy in Fig. 5b. The larger the molar fraction of Mg (green hexagons), Li (black pluses), and Be (blue triangles), the higher the formation energy of AlBeMgTiLi. In supplementary Fig. S5b–d, the color of the markers of the most stable compositions is primarily blue, indicating the low molar fraction of Mg, Be and Li. The most energetically stable composition of AlBeMgTiLi (Table 1) contains the highest molar fraction of Al (35%) and the lowest of Be (5%). The five topmost energetically stable compositions of AlBeMgTiLi are listed in Supplementary Table S6.

#### Mass density

The mean mass density of AlBeMgTiLi as a function of molar fraction of its constituent elements is shown in Fig. 5c. The largest negative slope of the mean density is for Li (black crosses), the lightest constituent element in AlBeMgTiCu. Li reduces the mass density to a greater extent than any other constituent element. Both Mg (green triangles) and Al (red circles) are characterized by smaller negative slopes, while the slope for Be (blue triangles) is nearly negligible. The large positive slope for Ti (yellow diamonds) implies that the mass density increases proportionally to its molar fraction.

In Fig. 5a, where the marker color indicates the molar fraction of Al, the compositions with the lowest mass density (see the region around yellow diamonds) contain the highest molar fraction of Al: The color of the markers is dark red. In a similar way, the marker color is exclusively red for the molar fraction of Li in Fig. S5b. In contrast to this, in Fig. S5d, where the marker color indicates the molar fraction of Ti, the compositions with the lowest mass density contain the smallest molar fraction of Ti: The color of markers is exclusively dark blue. Likewise, the color of the markers is mainly blue for Be (Fig. S5a), even though it is one of the lightest elements in AlBeMgTiLi. The reason is that Be significantly increases the formation energy (especially at its high molar fraction, shown in Fig. 5b), making most of AlBeMgTiLi compositions unstable. Therefore, the lightest stable compositions contain a comparatively low molar fraction of Be. As can be seen in Fig. S5c, where the marker color indicates the molar fraction of Mg, the compositions with the lowest density may contain any molar fraction of Mg: A combination of the blue and red colored markers represents these compositions. We found that the lightest stable composition of AlBeMgTiLi (Table 2) contains the highest molar fraction of Li and Al (32%) and the lowest of Be $($8%). The five topmost stable compositions of AlBeMgTiLi with the lowest mass density are reported in Supplementary Table S7.

#### Elastic modulus

**Figure 6:**
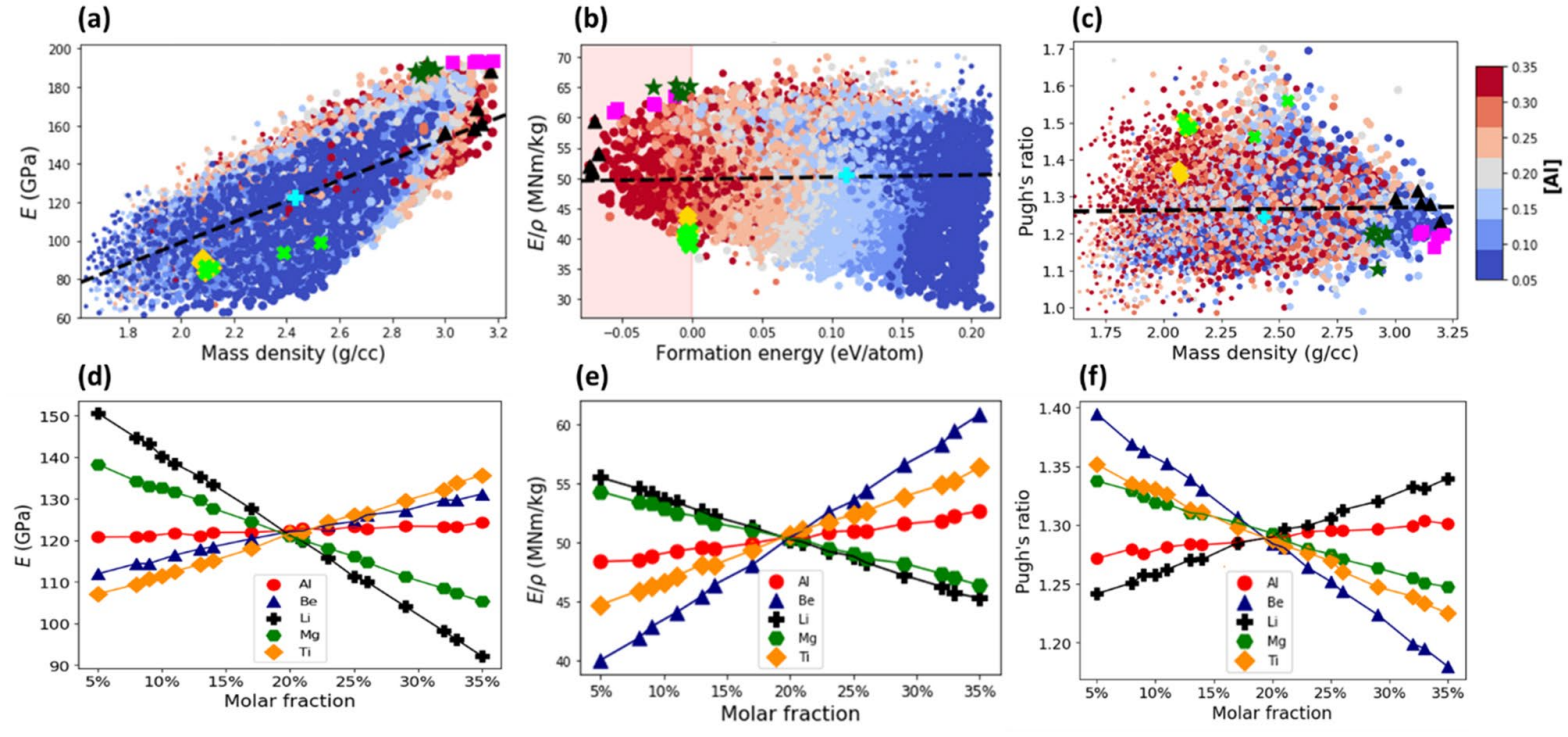
(**a**) The Young’s modulus versus the mass density for the grid compositions of AlBeMgTiLi with BCC lattice in single SS phase. (**b**) The specific stiffness versus the formation energy. The shaded region contains the stable compositions only (**c**) The Pugh’s ratio versus the mass density. In (**a**–**c**) the marker color indicates the molar fraction of Al as shown by the side colorbar and the marker size is proportional to the molar fraction of Ti. The dashed line indicates correlation between the elastic modulus and density in (**a**), specific stiffness and formation energy in (**b**), and Pugh’s ratio and density in (**c**). The topmost stable compositions with the lowest energy indicated by black triangles, with the lowest mass density by yellow diamonds, with the highest elastic modulus by magenta squares, with the highest specific stiffness by green stars and with the largest Pugh’s ratio by green crosses. The equimolar composition is specified by cyan plus marker. (**d**–**e**) The mean elastic modulus (**d**), specific stiffness (**e**) and Pugh’s ratio (**f**) as a function of molar fraction of its constituent elements.

Figure 6a plots the elastic modulus versus the mass density for all the compositions of AlBeMgTiLi. Like in the case of AlBeMgTiSi HEA, the data points are arranged in an elliptically shaped pattern. The major axis of the pattern is oriented along the dashed line. The positive slope of the line suggests a positive linear correlation between the $E$ and $\rho$: the bigger the mass density, the larger the elastic modulus. In Fig. 6b, we indicate the topmost energetically stable compositions (black triangles), the lightest compositions (yellow diamonds), the compositions with the highest Pugh’s ratio (green crosses), elastic modulus (magenta squares) and specific stiffness (green stars). The different groups of the topmost compositions are separated from each other. Therefore, it is impossible to identify AlBeMgTiLi compositions, which are all together the most energetically stable, lightweight, ductile and of the highest elastic modulus and specific stiffness.

The mean elastic modulus as a function of the molar fraction of the constituent elements is shown in Fig. 6d. The large positive slopes of Ti (yellow diamonds), Be (blue triangles), and Al (red circles) indicate that the higher their molar fraction, the larger the elastic modulus. The molar fraction of Al is shown by the marker color in Fig. 6a: The stable compositions with the largest $E$ contain the high molar fraction of Al since the color of the markers at the top right corner is predominantly red. Likewise, in Supplementary Fig. S6a for Be (and Fig. S6b for Ti), the color of the markers for the compositions with the highest $E$ is red. In contrast, the large negative slopes for Li (black crosses in Fig. 6d) and Mg (green hexagons) imply that $E$ can be significantly reduced by an increase in their molar fraction. As shown in Fig. S6c for Li and Fig. S6d for Mg, the color of the markers for the compositions with the highest $E$ is exclusively dark blue. The top stable composition of AlBeMgTiLi with the highest $E$ (Table 3) contains the highest molar fraction of Be and Ti (32%) and the lowest of Li and Mg (5%). The five topmost stable AlBeMgTiLi compositions with the highest elastic modulus are reported in Supplementary Table S8.

#### Specific stiffness

Figure 6b plots the specific stiffness versus the formation energy for all the compositions of AlBeMgTiLi. As can be seen in Fig. 6b, the *E/ρ* values of the topmost energetically stable compositions (black triangles) are closer to the average ones, while those with the lowest mass density (yellow diamonds) are closer to the lowest value of $E/\rho$. The compositions with the highest $E$ (magenta squares) partially overlap with those with the highest $E/\rho$ (green stars).

An increase in the molar fraction of Be (blue triangles), Al (red circles) and Ti (yellow diamonds) significantly increases $E/\rho$, while an increase in the molar fraction of Mg (green hexagons) and Li (black crosses) reduces $E/\rho$ (Fig. 6e). The molar fraction of Al is specified by the marker color in Fig. 6b: The stable compositions with the high $E/\rho$ values (see the area near black triangles) contain the highest molar fraction of Al since the color of the markers is entirely dark red. In a similar fashion, the red marker color in Supplementary Fig. S7a for Be and Fig. S7b for Ti indicates that the compositions with the high $E/\rho$ values contain the large molar fraction of Be and Ti. By contrast, the predominantly blue marker color in supplementary Fig. S7c for Mg (and Fig. S7d for Li) implies that the compositions with high $E/\rho$ values contain low molar fractions of these elements. We found that the stable composition of AlBeMgTiSi with the highest specific stiffness (Table 4) contains the highest molar fraction of Be and Ti (33%) and the lowest of Mg and Li (5%). The five topmost stable compositions with the highest *E/ρ* values are reported in Supplementary Table S9.

#### Pugh’s ratio

Figure 6c plots the Pugh’s ratio versus the mass density for all the compositions of AlBeMgTiLi. Like in the case of AlBeMgTiSi, all the AlBeMgTiLi compositions are brittle. Figure 6f plots the mean value of Pugh’s ratio for AlBeMgTiLi as a function of the molar fraction of its constituent elements. An increase in the molar fraction of Li and Al increases $B/G$, while the effect of Mg, Ti and especially Be is negative: The higher their molar fraction, the more brittle the given composition.

As can be seen in Fig. 6c, where the marker color indicates the molar fraction of Al, the compositions with the highest Pugh’s ratio may contain almost any molar fraction of Al: The color of the markers varies from blue to red, although it is predominantly red at the low-density region. Similarly, the mixed marker color distribution characterizes the weak effect of molar fraction of Li, Mg, and Ti in Supplementary Fig. S8b–d. By contrast, in Fig. S8a, the color of the markers for the compositions with the highest Pugh’s ratio is primarily blue, representing the lowest molar fraction of Be. We found that the top stable AlBeMgTiLi composition with the highest Pugh’s ratio (Table 5) contains the largest molar fraction of Al (32%) along with the smallest fraction of Be (5%). The five topmost stable AlBeMgTiLi compositions with the highest Pugh’s ratio are listed in Supplementary Table S10.

### AlBeMgTiCu

#### Formation energy

**Figure 7:**
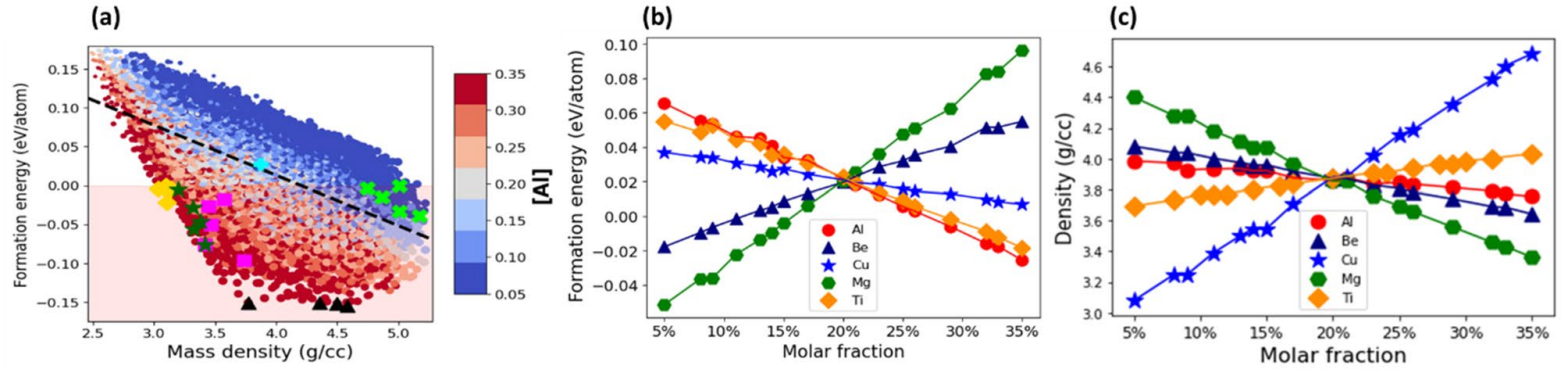
(**a**) The formation energy per atom versus the mass density for all the grid compositions of AlBeMgTiCu with BCC lattice in single SS phase. The color of the markers indicates the molar fraction of Al as shown by the side colorbar and the marker size is proportional to the molar fraction of Ti. The shaded region includes the energetically stable compositions only. The topmost stable compositions with the lowest energy indicated by black triangles, with the lowest mass density by yellow diamonds, with the highest elastic modulus by magenta squares, with the highest specific stiffness by green stars and with the largest Pugh’s ratio by green crosses. The equimolar composition is specified by cyan plus marker. The dotted line indicates correlation between the formation energy and mass density. (**b**, **c**) The mean formation energy (**b**) and mass density (**c**) as a function of molar fraction of its constituent elements. The mean values are calculated as an average over the compositions with a given molar fraction of a constituent element.

Figure 7a plots the formation energy per atom versus the mass density for all the grid compositions of AlBeMgTiCu. The data points form a droplet-like pattern. The dashed line with the negative slope indicates a negative correlation between the formation energy and the mass density: The larger the density of a given composition, the lower the formation energy and the greater its stability.

As can be seen in Fig. 7a, the topmost energetically stable compositions are in the density range between ρ = 4.4 g/cm^3^ and ρ = 4.7 g/cm^3^ (black triangles). They are separated from the topmost lightest stable compositions located between ρ = 3 g/cm^3^ and ρ = 3.4 g/cm^3^ (yellow diamonds). The topmost stable compositions with the highest $E/\rho$ (green stars) are adjacent to the topmost lightweight compositions, which is favorable for the selection of stable lightweight compositions with high specific stiffness. The topmost compositions with the highest $E$ are located between ρ = 3.5 g/cm^3^ and ρ = 4.1 g/cm^3^ (magenta squares), while the topmost stable compositions with the highest Pugh’s ratio are scattered (green crosses). Thus, the five groups of the topmost AlBeMgTiCu compositions selected according to the different design criteria are found in the distinct locations in the formation energy—mass density plane. Therefore, a single composition satisfying all the five criteria is nonexistent, and thus one must rank the selection criteria to find an appropriate practical solution according to the specific ranking.

The region with the most stable compositions is around the topmost energetically stable compositions (black triangles in Fig. 7a). As shown in Fig. 7a, these compositions contain the highest molar fraction of Al (as shown by the dark red colored markers). This is also indicated by the large negative slope of the mean formation energy plotted versus the molar fraction of Al (red circles in Fig. 7b). Similarly, an increase in the molar fraction of Ti (yellow diamonds) and Cu (blue stars) lowers the formation energy of AlBeMgTiCu. The most stable compositions of AlBeMgTiCu contain the highest molar fraction of Ti (red markers near black triangles in Supplementary Fig. S9a). By contrast, the presence of a small fraction of the blue markers among the red ones in Fig. S9b suggests that a few compositions with low molar fractions of Cu are also highly stable. An increase in the molar fraction of Be (blue triangles in Fig. 7b) and Mg (green hexagons) raises the formation energy of AlBeMgTiCu as indicated by their large positive slopes. As shown in Supplementary Fig. S9c for Mg and Fig. S9d for Be, the most energetically stable compositions marked by dark blue contain the lowest molar fraction of Be and Mg. We found that the most energetically stable AlBeMgTiCu composition (Table 1) includes the highest molar fraction of Ti (35%), and the lowest of Mg and Be (5%). The five topmost energetically stable AlBeMgTiCu compositions are reported in Table S11.

#### Mass density

Figure 7c plots the mean mass density of AlBeMgTiCu as a function of the molar fraction of its constituent elements. The largest negative slope of the mean formation energy is for Be (blue triangles), which is the lightest constituent element in AlBeMgTiCu: it significantly reduces the mass density. The intermediate negative slopes for Al (red circles) and Mg (green hexagons) are similar since the atomic masses of these elements are comparable. By contrast, the largest positive slopes for Cu (blue stars) and Ti (yellow diamonds) show that the mass density increases with an increase in their molar fraction. The effect is remarkably strong for Cu, the heaviest constituent element in AlBeMgTiCu.

In Fig. 7a, where the marker color indicates the molar fraction of Al, the compositions with the lowest density (the area near yellow diamonds) contain the highest molar fraction of Al: The color of the markers is exclusively dark red. In a similar fashion, the marker color is predominantly red for the molar fraction of Ti in these compositions (Supplementary Fig. S9a). By contrast, in Fig. S9b, where the marker color indicates the molar fraction of Cu, the compositions with the lowest mass density contain the lowest molar fraction of Cu: The marker colors are entirely dark blue. The marker color is also blue for Be (see Fig. S9d). This constituent element increases the formation energy (Fig. 7b), making AlBeMgTiCu more unstable. Therefore, the stable, lightest AlBeMgTiCu compositions contain the low molar fraction of Be. Like Be, Mg also increases the formation energy (Fig. 7b), making AlBeMgTiCu less stable. Yet at the moderate molar fraction of Mg, the compositions with the lowest density are stable (supplementary Fig. S9d): The color of the markers is predominantly light red. We found that the lightest stable composition of AlBeMgTiCu (Table 2) contains the highest molar fraction of Al (35%) and the lowest of Cu (5%). The five topmost stable lightweight compositions of AlBeMgTiCu are reported in Supplementary Table S12.

#### Elastic modulus

**Figure 8:**
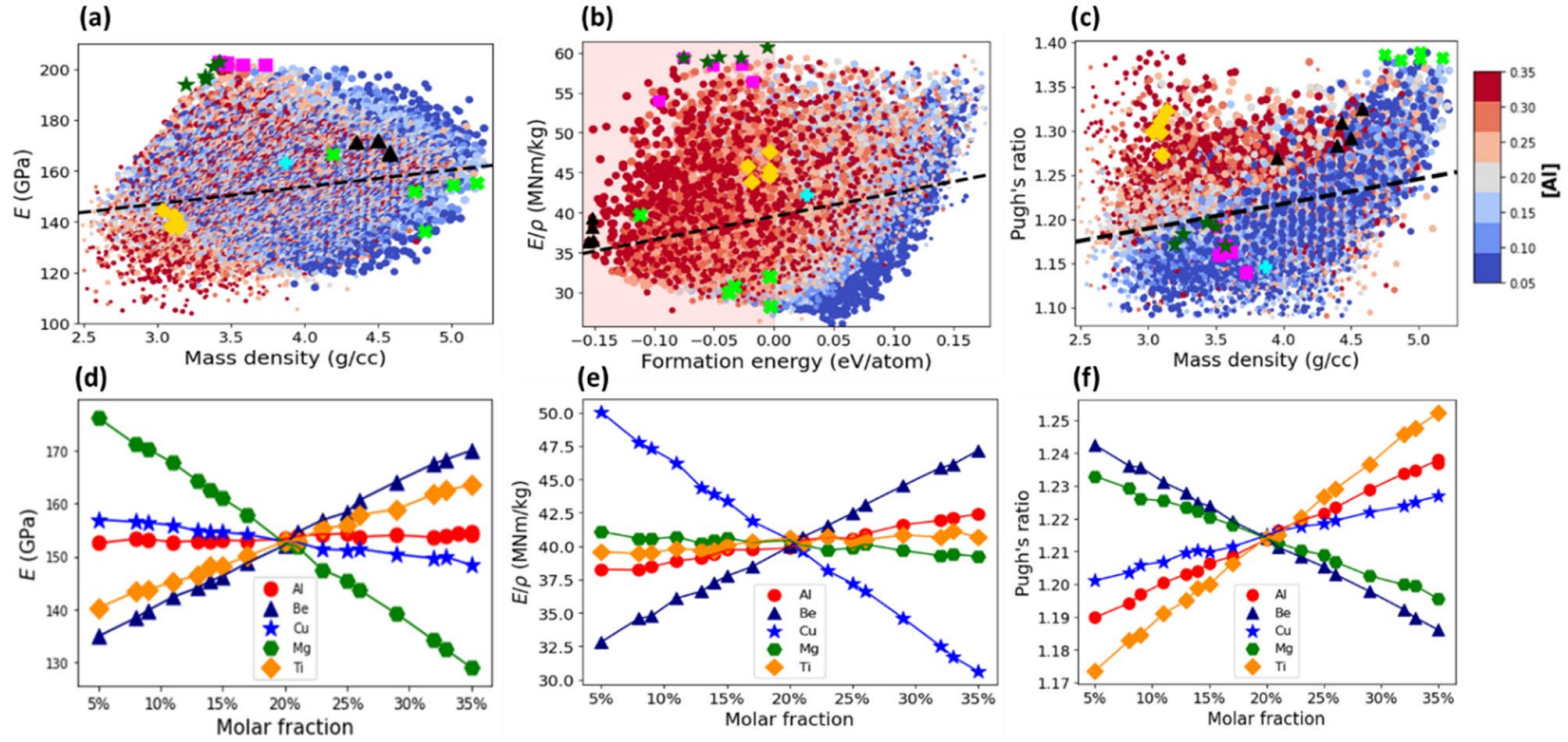
(**a**) The Young’s modulus versus the mass density for all the grid compositions of AlBeMgTiCu with BCC lattice in single SS phase. (**b**) The specific stiffness versus the formation energy. The shaded region contains only the stable compositions. (**c**) The Pugh’s ratio versus the mass density. The marker color indicates the molar fraction of Al as shown by the side colorbar and the marker size is proportional to the molar fraction of Ti. The dashed line indicates the correlation between the elastic modulus and density in (**a**), specific stiffness and formation energy in (**b**), and Pugh’s ratio and density in (**c**). The topmost stable compositions with the lowest energy indicated by black triangles, with the lowest mass density by yellow diamonds, with the highest elastic modulus by magenta squares, with the highest specific stiffness by green stars and with the largest Pugh’s ratio by green crosses. The equimolar composition is specified by cyan plus marker. The mean elastic modulus (**d**), specific stiffness (**e**) and Pugh’s ratio (**f**) as a function of molar fraction of its constituent elements.

Figure 8a plots Young’s modulus versus the mass density for all the AlBeMgTiCu compositions. As can be seen in Fig. 8a, the compositions with the largest elastic modulus (magenta squares) and specific stiffness (green stars) are in the medium mass density range, while the topmost energetically stable compositions (black triangles) are in the higher density region, and the topmost lightweight compositions (yellow diamonds) are in the lower density region. The topmost stable ductile compositions can be found in both the low- and high-density regions (green crosses). The five groups of the topmost composition are separated, causing difficulties in finding an AlBeMgTiCu composition which is all together stable, most lightweight, most ductile and of the highest Young’s modulus and specific stiffness.

Figure 8d plots the mean elastic modulus of AlBeMgTiCu as a function of the molar fraction of its constituent elements. Apparently, the higher the molar fraction of Be (blue triangles) and Ti (yellow diamonds), the larger Young’s modulus. The molar fraction of Be is shown by the marker color in Supplementary Fig. S10a: The stable compositions with the largest $E$ contain the highest molar fraction of Be since the color of the markers is dark red. Likewise, in Supplementary Fig. S10b for Ti, the color of the markers for the compositions with the highest $E$ is mostly red. The relatively small slopes of Al (red circles in Fig. 8d) and Cu (blue stars in Fig. 8d) imply that $E$ is less affected by these elements. The marker colors indicating the molar fraction of these constituent elements (Fig. 8a for Al and supplementary Fig. S10c for Cu) in the composition with the high $E$ values can be red or blue. On the contrary, the large negative slope for Mg (green hexagons in Fig. 8d) indicates that an increase in its molar fraction substantially reduces $E$. The molar fraction of Mg is indicated by the marker color in Supplementary Fig. S10d: The stable compositions with the largest $E$ contain the lowest molar fraction of Mg since the color of the markers is dark blue. The stable composition of AlBeMgTiCu with the largest $E$ value (Table 3) contains the highest molar fraction of Be and Ti (32%), and the lowest of Cu and Mg (5%). The five topmost stable AlBeMgTiCu compositions with the highest elastic modulus are listed in Supplementary Table S13.

#### Specific stiffness

Figure 8b plots the specific stiffness versus the formation energy for all the compositions of AlBeMgTiCu. As can be seen in Fig. 8b, the topmost energetically stable compositions (black triangles), the most lightweight compositions (yellow diamonds), and compositions with the highest $E$ (magenta squares) or $E/\rho$ (green stars) are separated.

The molar fraction of Al in each composition is specified by the marker color in Fig. 8b: The stable compositions with the high $E/\rho$ values, located in the vicinity of the topmost compositions with the highest $E/\rho$ values (green stars), are red colored with the moderate to high molar fraction of Al. As shown in Fig. 8e, the positive slope of the mean $E/\rho$ as a function of molar fraction of Al (red circles) and Ti (yellow diamonds) indicates that an increase in the molar fraction of Al and Ti moderately increases *E/ρ.* The much greater slope for Be (blue triangles) implies that its effect is considerably stronger. In supplementary Fig. S11a, the marker color of the compositions with the high $E/\rho$ value is exclusively dark red, indicating the highest molar fraction of Be. By contrast, the large negative slope for Cu (blue stars in Fig. 8e) indicates that it can significantly reduce $E/\rho$. In supplementary Fig. S11b, the marker color for the compositions with the high $E/\rho$ value is entirely dark blue, indicating the lowest molar fraction of Cu. Similarly, the negative slope for Mg (green stars in Fig. 8e) shows that an increase in its molar fraction reduces $E/\rho$, although the effect is significantly weaker than that of Cu. In Supplementary Fig. S11c, the blue marker color indicates the low molar fraction of Mg, while the red one implies the high molar fraction of Ti in Fig. S11d in the compositions with the high specific stiffness. We found that that the top stable AlBeMgTiCu composition with the largest specific stiffness (Table 4) contains the highest molar fraction of Be (35%) and the lowest of Cu (5%). The five topmost stable compositions with the highest *E/ρ* are listed in Supplementary Table S14.

#### Pugh’s ratio

Figure 8c plots the Pugh’s ratio versus the mass density for all the compositions of AlBeMgTiCu. The compositions are brittle according to the criterion of Pugh. The mean Pugh’s ratio as a function of the molar fraction of its constituent elements is shown in Fig. 8f. Noticeably, the positive slopes of Ti, Al, and Cu suggest that an increase in their molar fraction increases on average the Pugh’s ratio, while the effect of Be and Mg is the opposite.

As shown in Fig. 8c, where the marker color indicates the molar fraction of Al, the compositions with the highest Pugh’s ratio may contain the high or low molar fraction of Al: The marker color varies between blue and red in the region near the topmost compositions with the highest Pugh’s ratio (green crosses). The molar fraction of Ti is indicated by the marker color in supplementary Fig. S12a: The stable compositions with the high $B/G$ ratio contain the high molar fraction of Ti since the color of the markers is mostly red. For Cu and Mg, the compositions with the high Pugh’s ratio may contain high or low molar fractions of these elements (Supplementary Fig. S12b,c). By contrast, the color of the markers is primarily dark blue in Fig. S12d, indicating the low molar fraction of Be. We found that the top stable AlBeMgTiCu composition with the highest Pugh’s ratio (Table 5) contains the largest molar fraction of Ti (35%) and the smallest of Be (6%). The five topmost stable compositions of AlBeMgTiCu with the highest Pugh’s ratio are listed in Supplementary Table S15.

## Discussion

### Energetical stability

Our DFT calculations are performed at 0 K for the ground state of these lightweight HEAs. This brings up an important question: How stable are the identified topmost compositions (with the lowest formation energy) in SS state against their compound phases at room temperature? To answer the question, we used the Grand Canonical Linear Programming (GCLP) method developed by Wolverton et al.^56^ for metallic compounds. The method maps a free energy minimization problem to a linear algebra problem to predict the free energy minimum for a given composition at room temperature. We compared the formation energy for the most stable HEA compositions obtained at T = 0 K and room temperature (T = 300 K). At room temperature, we added a term corresponding to the configurational entropy of mixing, $TS={-Tk}_{B}\sum_{i}{n}_{i}log({n}_{i})$, to the free energy. The results are reported in Table 1.

As can be seen in Table 1, only AlBeMgTiSi is energetically stable against compound segregation. AlBeMgTiCu and AlBeMgTiLi in single SS state are unstable against compound segregation, hence specialized processing routes are required to prepare such lightweight HEAs: for example, one may freeze AlBeMgTiCu and AlBeMgTiLi in a metastable SS state via fast cooling.

Among the three studied HEA families, four principal elements (Al, Be, Mg, and Ti) are the same, and one element is different (Cu, Si, and Li). Here, we examine how the substitution of one of the constituent elements by another, for instance, Cu by Si or Li, affects the formation energy and other physical properties of the lightweight HEAs.

As shown in Table 1, AlBeMgTiSi has the lowest formation energy, which is considerably lower than that of AlBeMgTiCu and AlBeMgTiLi. This is due to the exceptional stabilizing role of Si, especially at its high molar fraction (Fig. 3b). Replacement of Cu by Si reduces almost four-fold the formation energy of the most stable composition AlBeMgTiSi as compared to that of AlBeMgTiCu. While substituting Cu with Li increases the formation energy of AlBeMgTiLi.

To qualitatively explain the effect of constituent element substitutions, we selected a set of atomic descriptors, which correlate with the formation energy: The enthalpy of mixing ($\Delta {H}_{mix}$), cohesive energy (${E}_{coh}$), valence electron concentration ($VEC$), and electronegativity ($\chi$). The effect of each constituent element is considered by taking into account its contribution to a chosen atomic descriptor.

We find that $\Delta {H}_{mix}$ and ${E}_{coh}$ are markedly correlated with the formation energy as shown in Supplementary Fig. S13. By its nature, $\Delta {H}_{mix}$ describes pair interaction between the different types of constituent elements, while ${E}_{coh}$ describes pair interaction between the same type of constituent elements. The stronger the interaction between the constituent elements, the lower the formation energy, and therefore, the more stable the given HEA composition.

We calculated $\Delta {H}_{mix}$ according to the semi-empirical model of binary alloy cohesion developed by Miedema et al.^57^: $\Delta {H}_{mix}=4\sum_{i}\sum_{j>i}\Delta {H}_{ij}{c}_{i}{c}_{j}$ , where $\Delta {H}_{ij}$ is the enthalpy of mixing between an $ij$ pair of the constituent elements and ${c}_{i}$ is the molar fraction of the $i$th constituent element. As can be seen in Supplementary Fig. S13a–c, the smaller $\Delta {H}_{mix}$, the lower is the formation energy. We calculated the mean enthalpy of mixing between Cu (Si and Li) and the remaining four elements (Al, Be, Mg and Ti) to estimate its contribution to the $\Delta {H}_{mix}$. For example, the mean enthalpy of mixing for Si: $<\Delta {H}_{mix,Si}>=1/4(\Delta {H}_{Si,Al}+\Delta {H}_{Si,Be}+\Delta {H}_{Si,Ti}+\Delta {H}_{Si,Mg})$. Since $<\Delta {H}_{mix,Si}>$ for Si (-31.5 kJ/mol) is significantly smaller than that of Cu (-3.3 kJ/mol), and the molar fraction of Si (35%) for the most stable composition is larger than that of Cu (5%), the exchange of Cu by Si reduces the formation energy. It should be noted that Si may increase alloy stability by forming ionic or covalent bonds to other metals^58^, rather than like Li or Cu, which forms metallic bonds. By contrast, the mean enthalpy of mixing for Li (6.3 kJ/mol) is larger than that of Cu, and its molar fraction of Li (14%) for the most stable composition is larger than that of Cu, thus the exchange of Cu by Li increases the formation energy.

The cohesive energy $<{E}_{coh}>$ = $\sum_{i}{E}_{coh,i}{c}_{i}$ of each composition was calculated by the rule of mixtures using the cohesive energies ${E}_{coh,i}$ (for their most stable crystalline phases) and molar fraction ${c}_{i}$ of the constituent elements. The cohesive energy is plotted against the formation energy in Supplementary Fig. S13d–f. According to our DFT calculations, the cohesive energies of Cu, Si and Li are ${E}_{coh,Cu}$ = 336 kJ/mol,${E}_{coh,Si}$ = 447 kJ/mol and ${E}_{coh, Li}$ = 157 kJ/mol, respectively. Since the formation energy is positively correlated with the cohesive energy, the exchange of Cu by Si reduces the formation energy, while the substitution Cu by Li increases it.

Next, we calculated $$VEC$$ for every composition by the rule of mixtures: $$VEC=\sum_{i}{c}_{i}{[VEC]}_{i},$$ where $${[VEC]}_{i}$$ is the valence electron concentration of the $$i$$th constituent element and $${c}_{i}$$ is its molar fraction. As can be seen in Supplementary Fig. S14a–c, the higher the $$VEC$$ value, the lower the formation energy. The $$VEC$$ values of the constituent elements are taken from literature^59^: Since $$VEC$$ of Si ($$VEC=4)$$ is larger than that of Cu or Li ($$VEC=1$$), and the molar fraction of Si is the highest for the most stable composition, the replacement of Cu by Si thus lowers the formation energy.

The Pauling electronegativity is also calculated by using the rule of mixtures: $<\chi >=\sum_{i}{c}_{i}{\chi }_{i}$, where ${\chi }_{i}$ is the electronegativity and ${c}_{i}$ is the molar fraction of the $i$th constituent element. We note that $<\chi >$ is an important atomic descriptor for the charge transfer effects^60^, and therefore for the formation energy. The higher the $<\chi >$ value, the lower the formation energy (supplementary Fig. S14d–f). The electronegativities of the constituent elements are taken from literature^61^. The electronegativities of Si and Cu are the same ($\chi$ = 1.9), while that of Li ($\chi$ = 0.9) is smaller. Since the average electronegativity over the four elements (Al, Be, Mg and Ti) is ${\chi }_{ave}=1.5$, the substitution of Cu by Li reduces $<\chi >$ , and consequently increases the formation energy.

### Mass density

The lightest stable compositions of the examined HEAs are listed in Table 2. As can be expected, AlBeMgTiLi containing the two lightest constituent elements Li and Be has the lowest density, while AlBeMgTiCu has the highest one. The mass density is significantly reduced when the relatively heavy Cu atoms are replaced by Si or Li. The density of the lightest AlBeMgTiSi composition is lower by ~ 20% than that of AlBeMgTiCu, while AlBeMgTiLi is by ~ 30%.

### Elastic modulus

In Table 3, we include the stable compositions with the highest elastic modulus sorted in a descending order. We found that the elastic modulus for the topmost AlBeMgTiSi composition is slightly larger than that of AlBeMgTiCu, which is higher than the elastic modulus of the best AlBeMgTiLi composition.

As can be seen in Table 3, the replacement of Cu by Si marginally increases the elastic modulus, while the substitution Cu by Li reduces it. To better understand the substitution effect, we examined the relation between the elastic modulus and the selected atomic descriptors, which (according to ML-based studies^62,63^) determine the value of Young’s modulus. The following atomic descriptors were considered: melting temperature, ${T}_{m}$, electronegativity mismatch, $\Delta \chi$, as well as $VEC$, $\Delta {H}_{mix}$ and ${E}_{coh}$.

The average ${T}_{m}$ is calculated according to the rule of mixtures ${T}_{m}=\sum_{i}{c}_{i}{T}_{m,i}$ by using the molar fractions, ${c}_{i},$ and melting temperatures ${, T}_{m,i},$ of the most stable crystalline phases of the constituent elements. As can be seen in Supplementary Fig. S15, an increase in the elastic modulus positively correlates with an increase in ${T}_{m}$. Roy et al.^63^ suggested that ${T}_{m}$ is the most important atomistic descriptor to predict the Young’s modulus since it can be considered as an implicit metric of bond strength (which determines the value of elastic modulus). Since ${T}_{m}$ of Si (1683 K)^64^ is higher than that of Cu (1358 K)^64^, the substitution of Cu by Si increases the value of Young’s modulus. By contrast, ${T}_{m}$ of Li (454 K)^64^ is substantially smaller than that of Cu, hence the substitution of Cu by Li reduces the elastic modulus.

As can be seen in Supplementary Fig. S16a–c, the value of elastic modulus rises with increasing $$VEC$$. In metallic alloys, the value of Young’s modulus is determined by the resistance of the valence electron gas to compression. Consequently, the larger the VEC value, the greater the resistance to compression, and thus the higher the elastic modulus^62^. Since $$VEC$$ of Si ($$VEC=4)$$ is greater than that of Cu ($$VEC=1$$), and the molar fraction of Si is the highest in the compositions with the largest elastic modulus, the replacement of Cu by Si increases the Young’s modulus.

The Pauling electronegativity mismatch^60^ $\Delta \chi =\sqrt{\sum_{i}{c}_{i}{\left(1-\frac{{\chi }_{i}}{<\chi >}\right)}^{2}}$ is calculated as a mean squared deviation of the individual electronegativities of the constituent elements ${\chi }_{i}$ from the average $<\chi >$ weighted by their molar fraction ${c}_{i}$. The value of Young’s modulus increases with decreasing $\Delta \chi$ (Supplementary Fig. S16d–f). The smaller the $\Delta \chi$ mismatch, the more homogeneous the electron gas density, hence, the larger the electron gas resistance to compression, consequently the higher the Young’s modulus^65^. In addition, a substantial $\Delta \chi$ increases the probability of formation of brittle intermetallic phases^62^, thus reducing the elastic modulus. We note that the $\chi$ values of Si and Cu are equal ($\chi$ = 1.9) and relatively close to the mean ${\chi }_{av}=1.5$ of the remaining constituent elements (Al, Be, Mg and Ti), while that of Li ($\chi$ = 0.9) is smaller, which enhances $\Delta \chi$. Hence, the replacement of Cu by Li leads to a larger $\Delta \chi$, and therefore, reduces the Young’s modulus.

Both $\Delta {H}_{mix}$ and ${E}_{coh}$ are the essential atomic descriptors according to ML studies^62,63^ to predict the value of Young’s modulus. As can be seen in supplementary Fig. S16a–c, the smaller $\Delta {H}_{mix}$, the larger the Young’s modulus. Since the mean enthalpy of mixing between Si and the remaining four elements (− 31.5 kJ/mol) is significantly smaller than that of Cu (− 3.3 kJ/mol), the exchange of Cu by Si increases the Young’s modulus. By contrast, the mean enthalpy of mixing between Li and the remaining four elements (6.3 kJ/mol) is larger than that of Cu, thus the substitution of Cu by Li reduces the Young’s modulus. The cohesive energy is plotted against the Young’s modulus in supplementary Fig. S16d–f. It is apparent that the Young’s modulus increases with increasing cohesive energy. According to our DFT calculations, the cohesive energy of Si $($447 kJ/mol) is larger than that of Cu $($336 kJ/mol), and thus the exchange of Cu by Si enhances the elastic modulus, while the substitution Cu by Li $($157 kJ/mol) reduces the Young’s modulus.

### Specific stiffness

In Table 4, we report the stable compositions with the highest specific stiffness, $E/\rho ,$ sorted in a descending order. We found that the specific stiffness for AlBeMgTiSi and AlBeMgTiLi is higher than that of AlBeMgTiCu. The $E/\rho$ value is affected by the change in both $E$ and $\rho$. In the case of Cu replaced by Si, the two effects are acting in synergy: $E$ increases while $\rho$ decreases, and consequently $E/\rho$ of AlBeMgTiSi substantially increases (Table 4). In the case of Cu replaced by Li, both the $E$ and $\rho$ decrease. However, the reduction in $\rho$ is greater than that in $E$, and therefore $E/\rho$ of AlBeMgTiLi increases, although to a smaller degree (Table 4).

We also note that the $E/\rho$ values of the lightweight HEAs are markedly larger than those of HEAs with commonly used constituent elements: The low density of the lightweight HEAs leads to the higher specific stiffness. For example, the elastic modulus of AlCoCrFeNi^44^ is ${E}_{{\text{AlCoCrFeNi}}}/{E}_{{\text{AlBeMgTiSi}}}\approx$ 1.4 larger than that of AlBeMgTiSi, while its density is twice as large ${\rho }_{{\text{AlCoCrFeNi}}}/{\rho }_{{\text{AlBeMgTiSi}}}\approx$ 2, hence the $E/\rho$ of AlCoCrFeNi is only ≈ 0.7 of AlBeMgTiSi.

### Pugh’s ratio

Table 5 lists the stable compositions with the highest Pugh’s ratio. The topmost compositions of the examined lightweight HEAs are brittle, as opposed to the ductile HEAs with generally utilized constituent elements^66^.

The Pugh’s ratio for the topmost stable composition of AlBeMgTiLi is the highest and that of AlBeMgTiSi is the lowest. ML-based studies^67,68^ indicate that $\chi$ and $\Delta \chi$ are the two most appropriate atomistic descriptors for prediction of the $B/G$ ratio. As can be seen in Supplementary Fig. S18a–c, the Pugh’s ratio increases with decreasing $\chi$, in agreement with the ML results by Mukhamedov et al.^67^ Since Li has the lowest Pauling electronegativity as compared to Cu or Si, the Pugh’s ratio for the topmost AlBeMgTiLi composition is higher than that of AlBeMgTiCu. Similarly, the Pugh’s ratio increases generally with increasing $\Delta \chi$ as shown in Supplementary Fig. S18d–f. Since $\Delta \chi$ increases when Cu is substituted by Li, the Pugh’s ratio for the topmost AlBeMgTiLi composition is higher than that of AlBeMgTiCu (Table 5). Replacement of Cu by Si leads to the formation of partial covalent bonding, which is intrinsically brittle in contrast to non-directional metallic bonds, therefore, the Pugh’s ratio^69^ for the topmost AlBeMgTiSi composition is lower than that of AlBeMgTiCu.

### Comparison with aluminum alloys and stainless steel

We compared the physical properties of the lightweight HEAs with those of pure aluminum, as well as with a couple of the most widely used aluminum alloys: AA 6061, which includes Mg and Si as the primary alloying elements, and AA 7075, which contains Zn as the primary alloying element, and stainless steel grade 304^70,72^.

**Figure 9:**
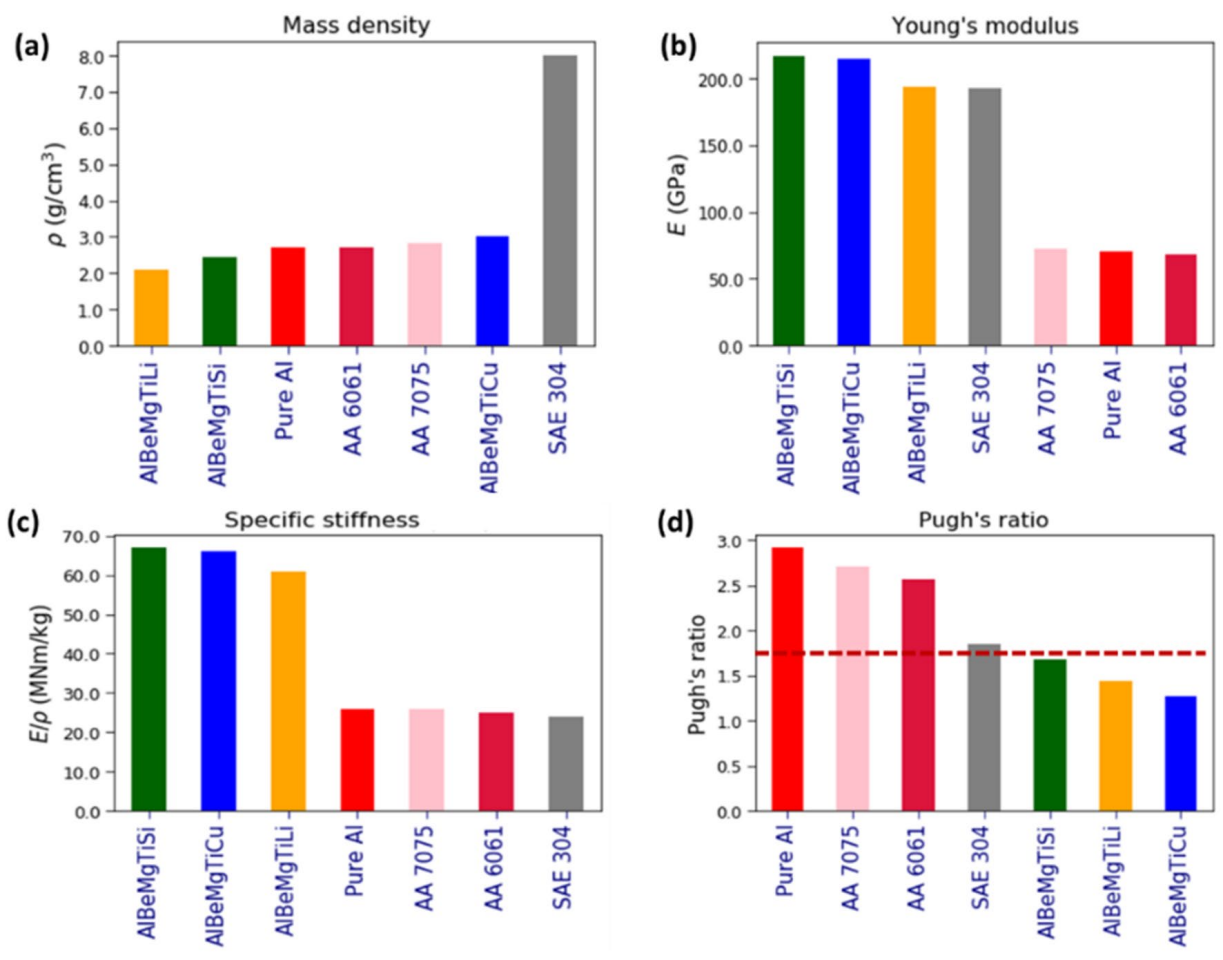
Comparison of the physical properties of the pure aluminum, selected aluminum alloys, and stainless steel with the examined lightweight HEAs.

The compiled data are shown in Table 6. As shown in Fig. 9, the examined lightweight HEAs have exceptional physical properties. First, they are the lightest alloys, with exclusion of AlBeMgTiCu, with densities lower than that of pure aluminum and its lightweight alloys, and much lower than that of the stainless steel (Fig. 9a). Apparently, this is the effect of inclusion of the lightweight principal elements, which drastically reduces the mass density. The elastic moduli of the lightweight HEAs are larger than that of the stainless steel, and substantially larger than those of pure aluminum and the aluminum alloys (Fig. 9b). Likewise, their specific stiffnesses are considerably larger than those of the stainless steel, pure aluminum, and the aluminum alloys (Fig. 9c). Yet, the according to the value of Pugh’s ratio, the lightweight HEAs are relatively brittle in contrast to the stainless steel, pure aluminum, and the aluminum alloys (Fig. 9d).

**Table 6:** The selected physical properties of pure aluminum, aluminum alloys, stainless steel and the lightweight HEAs.

Alloy	Mass density (g/cm^3^)	Young’s modulus (GPa)	Specific stiffness (MN/kg)	Pugh’s ratio
Pure Al	2.71	70	26	2.92
AA 6061	2.72	68	25	2.56
AA 7075	2.81	72	26	2.71
Stainless steel 304	8.13	193	24	1.85
AlBeMgTiSi	2.43	217	67	1.68
AlBeMgTiCu	3.03	215	66	1.27
AlBeMgTiLi	2.08	194	61	1.44

## Conclusions

We applied the recently developed high-fidelity, high-throughput PSSOS method^44^ to design lightweight HEAs in a single SS phase. Since it is a first principles-based method, it enables us to examine HEAs with practically any chemical composition. By comprehensively examining the entire compositional space of three lightweight HEA families, we identified the topmost compositions using five design criteria: (1) high energetical stability, (2) low mass density, (3) large Young’s modulus, (4) large specific stiffness, and (5) high Pugh’s ratio. We showed that the PSSOS method can be efficiently used for HEA design.

To design lightweight alloys, we explored three quinary HEAs containing the lightest constituent elements (Li and Be), along with light constituent elements (Al, Mg and Si), namely: AlBeMgTiLi, AlBeMgTiSi and AlBeMgTiCu in both FCC and BCC lattice structures. The composition space was represented by a four-dimensional fine-resolution grid containing $\sim$ 9000 compositions. By using the chosen design criteria, we found the topmost HEA compositions with the highest stability, lowest density, largest elastic modulus or specific stiffness, and highest Pugh’s ratio.

We found that AlBeMgTiSi is the most energetically stable HEA, followed by AlBeMgTiCu and AlBeMgTiLi. Si is the most stabilizing constituent element, which greatly reduces the formation energy. Because of Si, the formation energy of AlBeMgTiSi is the lowest among the studied HEAs. AlBeMgTiLi is the lightest among the examined HEAs, followed by AlBeMgTiSi and AlBeMgTiCu. It was found that Li, Be, Mg and Al reduce the density in any HEA that contains them, and AlBeMgTiLi includes all of them. Amongst the considered HEAs, AlBeMgTiSi is not only the most stable alloy but also the one with the largest elastic modulus and specific stiffness, together with the highest Pugh’s ratio. We found that both Be and Si increase the elastic modulus and specific stiffness in any HEA containing them. The exceptionally low mass densities of the lightweight HEAs explain the remarkably high values of specific stiffness in comparison to the traditional HEAs. It was found that all the studied lightweight HEAs are likely brittle. The examined trends were qualitatively explained using correlations between the physical properties and the selected atomic descriptors.

The topmost HEA compositions found according to the selected design criteria are different. Therefore, if the HEA design requires applying several criteria at once, the resulting composition must be a trade-off in the selection of the optimized properties: One property can be optimized with some sacrifice in others.

The present study proved the utility and ability of the PSSOS method in the HEA design. The findings revealed here can serve as a guideline for the experimental fabrication of lightweight HEAs, which should create a far-reaching impact on the development and application of lightweight HEAs.

### Supplementary Information

Supplementary Information.

## Data Availability

The raw/processed data required to reproduce these findings cannot be shared at this time as the data also forms part of an ongoing study. The python code that was used to obtain the findings of this study are available from the corresponding authors upon reasonable request.
