# Supplementary material for: First principles-based design of lightweight high entropy alloys
Source: Sci Rep. 2023 Dec 18;13:22549. doi: 10.1038/s41598-023-49258-z (PMC10728166; doi:10.1038/s41598-023-49258-z)
Supplement: Supplementary file 1 — Supplementary Information. [file 41598_2023_49258_MOESM1_ESM.docx]

# First principles-based design of lightweight high entropy alloys

Viacheslav Sorkin^1^ (email: sorkinv@ihpc.a-star.edu.sg), Zhi Gen Yu^1^, Shuai Chen^1,2^, Teck Leong Tan^1^, Zachary Aitken^1^, Yong-Wei Zhang^1^(email: zhangyw@ihpc.a-star.edu.sg)

^1^ Institute of High Performance Computing (IHPC), Agency for Science, Technology and Research (A*STAR), 1 Fusionopolis Way, #16-16 Connexis, Singapore 138632, Republic of Singapore, ^2^Materials Genome Institute, Shanghai University, Shanghai 200444, China

**Abstract**

## **AlBeMgTiSi**

Figure S1 shows the formation energy per atom vs. the mass density for all the grid compositions of AlBeMgTiSi with BCC lattice in SS phase.

### ***Formation energy***


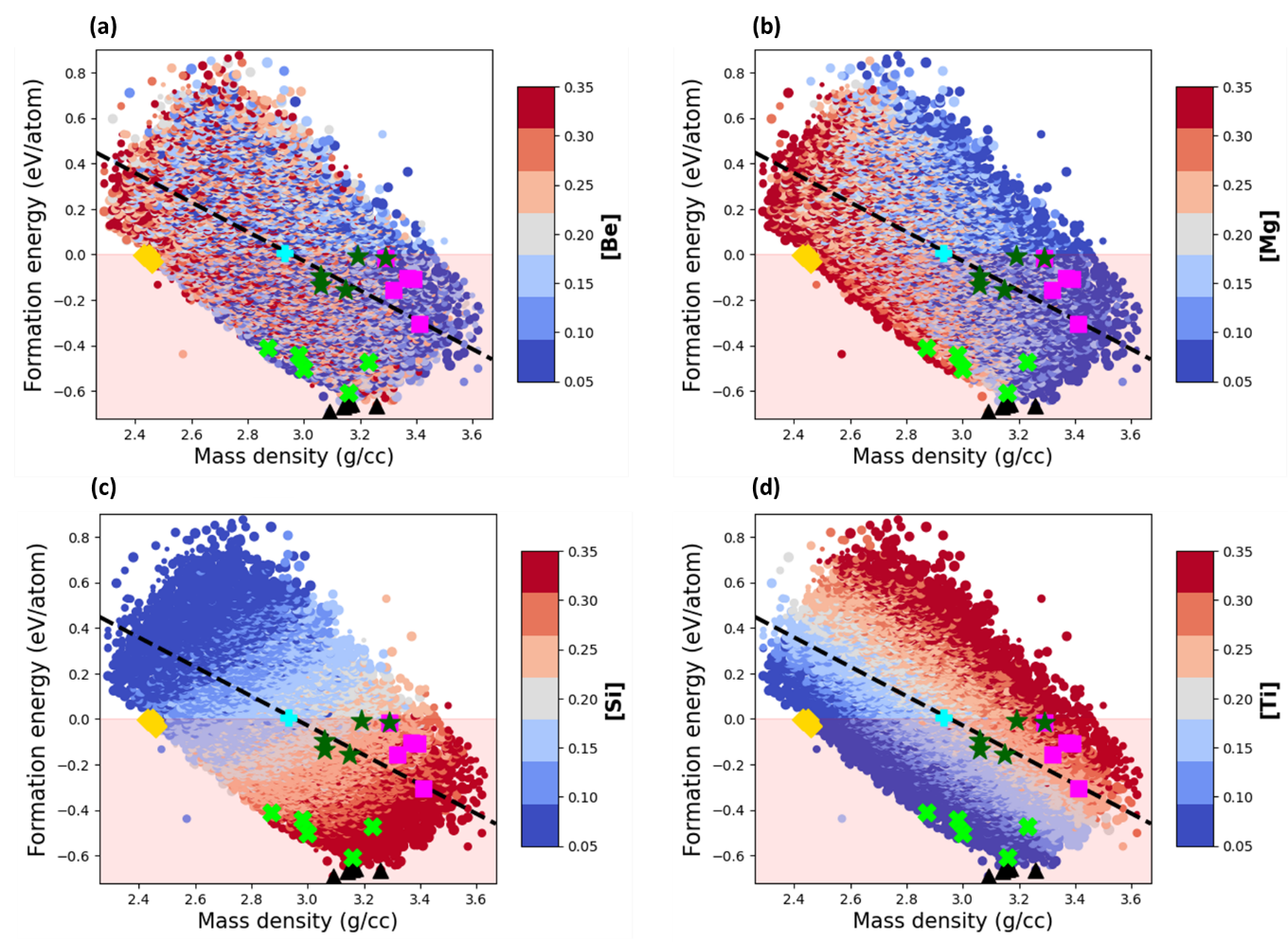


Figure S1: The formation energy per atom vs. the mass density for the compositions of AlBeMgTiSi with BCC lattice in SS phase. The black triangles correspond to the topmost energetically stable compositions, yellow diamonds represent the topmost stable compositions with lowest density, and green stars indicate ones with highest specific Young’s modulus. The magenta squares indicate the topmost stable compositions with the highest Young’s modulus and the topmost stable compositions with the highest Pugh’s ratio indicated by green crosses. The equimolar composition is specified by cyan plus marker. Dashed line indicates negative linear correlation between the formation energy and density. The marker color indicates molar fraction of Be (a), Mg (b), Si (c) and Ti (d) as shown by the side colorbar, and the marker size is proportional to the molar fraction of Al.

Table S1: The five topmost energetically stable compositions of the selected AlBeMgTiSi sorted in ascending order according to their values. The mass density, elastic modulus, specific stiffness and Pugh’s ration are reported for each composition.

| **Composition** | **Formation energy (eV/atom)** | **Mass density (g/cc)** | **Young’s modulus (GPa)** | **Specific Young’s modulus (MN/kg)** | **Pugh’s ratio (B/G)** |
| --- | --- | --- | --- | --- | --- |
| [0.31, 0.15, 0.14, 0.05, 0.35] | -0.691 | 2.43 | 135.77 | 55.90 | 0.95 |
| [0.29, 0.17,0.14, 0.05, 0.35] | -0.669 | 2.45 | 132.23 | 54.04 | 1.00 |
| [0.32, 0.14, 0.14, 0.05, 0.35] | -0.663 | 2.45 | 134.29 | 54.87 | 1.03 |
| [0.26, 0.23, 0.11, 0.05, 0.35] | -0.658 | 2.46 | 132.94 | 54.13 | 0.96 |
| [0.29, 0.21, 0.11, 0.05, 0.34] | -0.655 | 2.46 | 124.58 | 50.58 | 1.06 |

### ***Mass density***

Table S2: The most lightweight stable compositions of AlBeMgTiSi listed in an ascending order according to their values. In addition, we report the formation energy, elastic modulus, specific elastic modulus and Pugh’s ratio for each composition.

| **Composition** | **Mass density (****g/cc)** | **Formation energy (eV/atom)** | **Young’s modulus (GPa)** | **Specific Young’s modulus (MN/kg)** | **Pugh’s ratio (B/G)** |
| --- | --- | --- | --- | --- | --- |
| [0.11, 0.35, 0.35, 0.05, 0.14] | 2.43 | -0.003 | 135.77 | 55.90 | 0.95 |
| [0.17, 0.29, 0.35, 0.05, 0.14] | 2.45 | -0.011 | 132.23 | 54.04 | 1.00 |
| [0.14, 0.35, 0.32, 0.05, 0.14] | 2.45 | -0.004 | 134.29 | 54.87 | 1.03 |
| [0.23, 0.26, 0.35, 0.05, 0.11] | 2.46 | -0.031 | 132.94 | 54.13 | 0.96 |
| [0.29, 0.23, 0.32, 0.05, 0.11] | 2.46 | -0.027 | 124.58 | 50.58 | 1.06 |

### ***Elastic modulus***


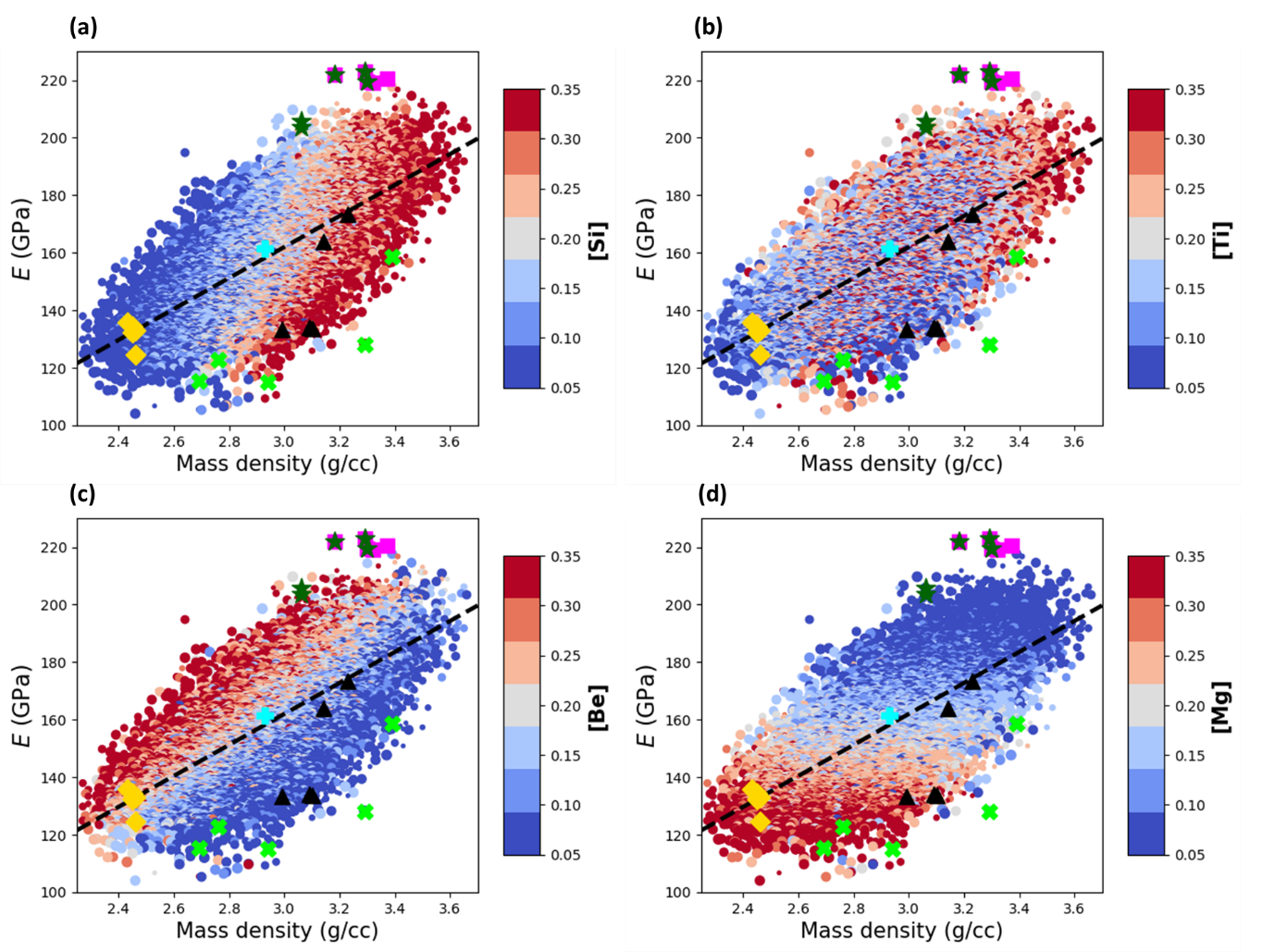


Figure S2: The elastic modulus vs. the mass density for the grid compositions of AlBeMgTiSi with BCC lattice in single SS phase. The black triangles correspond to the topmost energetically stable compositions, yellow diamonds represent the topmost stable compositions with lowest density, and green stars indicate ones with highest specific Young’s modulus. The magenta squares indicate the topmost stable compositions with the highest Young’s modulus and the topmost stable compositions with the highest Pugh’s ratio indicated by green crosses. Equimolar composition is specified by cyan plus marker. The dashed line indicates positive linear correlation between the Young’s modulus and mass density. The marker color indicates molar fraction of Si (a), Ti (b), Be (c) and Mg (d) as shown by the side colorbar, and the marker size is proportional to the molar fraction of Al.

Table S3: The stable compositions of AlBeMgTiSi with the largest Young’s modulus sorted in descending order according to their values. For each composition, we report the formation energy, mass density, specific elastic modulus and Pugh’s ratio.

| **Composition** | **Young’s modulus (GPa)** | **Formation energy (eV/atom)** | **Mass density (g/cc)** | **Specific Young’s modulus (MN/kg)** | **Pugh’s ratio (B/G)** |
| --- | --- | --- | --- | --- | --- |
| [0.26, 0.15, 0.05, 0.25, 0.29] | 222.85 | -0.017 | 3.29 | 67.78 | 0.87 |
| [0.33, 0.09, 0.05, 0.28, 0.25] | 220.52 | -0.102 | 3.37 | 65.38 | 0.79 |
| [0.05, 0.35, 0.05, 0.26, 0.29] | 219.03 | -0.156 | 3.32 | 65.98 | 0.88 |
| [0.29, 0.11, 0.05, 0.29, 0.26] | 217.61 | -0.109 | 3.39 | 64.20 | 1.00 |
| [0.08, 0.29, 0.05, 0.23, 0.35] | 216.75 | -0.306 | 3.41 | 63.58 | 0.91 |

### ***Specific elastic modulus***


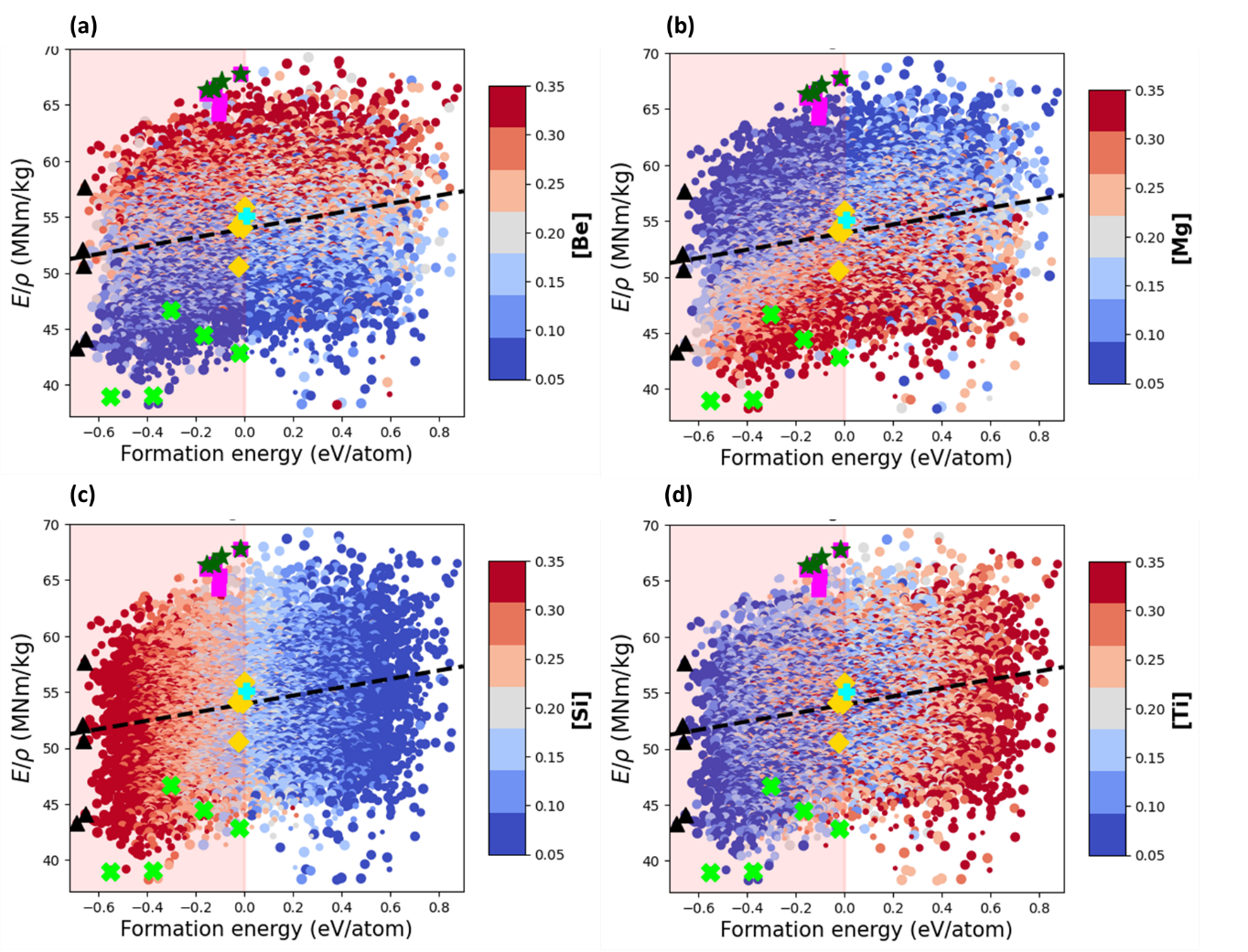


Figure S3: The specific elastic modulus vs. the formation energy per atom for the compositions of AlBeMgTiSi with BCC lattice in SS phase. The black triangles correspond to the topmost energetically stable compositions, yellow diamonds represent the topmost stable compositions with lowest density, and green stars indicate ones with highest specific Young’s modulus. The magenta squares indicate the topmost stable compositions with the highest Young’s modulus and the topmost stable compositions with the highest Pugh’s ratio indicated by green crosses. The equimolar composition is specified by cyan plus marker. The dashed line indicates positive correlation between the specific elastic modulus and formation energy. The marker color indicates molar fraction of Be (a), Mg (b), Si (c) and Ti (d) as shown by the side colorbar, and the marker size is proportional to the molar fraction of Al.

Table S4: The stable compositions of AlBeMgTiSi with the largest specific elastic modulus sorted in descending order according to their values. For each composition, we report the formation energy, mass density, elastic modulus, and Pugh’s ratio.

| **Composition** | **Specific stiffness (MN/kg)** | **Formation energy (eV/atom)** | **Mass density (g/cc)** | **Young’s modulus (GPa)** | **Pugh’s ratio (B/G)** |
| --- | --- | --- | --- | --- | --- |
| [0.05, 0.35, 0.05, 0.3, 0.25] | 67.78 | -0.017 | 3.29 | 222.85 | 0.87 |
| [0.35, 0.2, 0.08, 0.17, 0.2] | 67.19 | -0.096 | 3.06 | 205.75 | 0.86 |
| [0.25, 0.33, 0.05, 0.16, 0.21] | 66.47 | -0.132 | 3.06 | 203.52 | 0.89 |
| [0.15, 0.35, 0.05, 0.2, 0.25] | 66.37 | -0.155 | 3.15 | 208.83 | 0.88 |
| [0.05, 0.35, 0.08, 0.29, 0.23] | 66.36 | -0.006 | 3.19 | 211.83 | 0.85 |

### ***Pugh’s ratio***


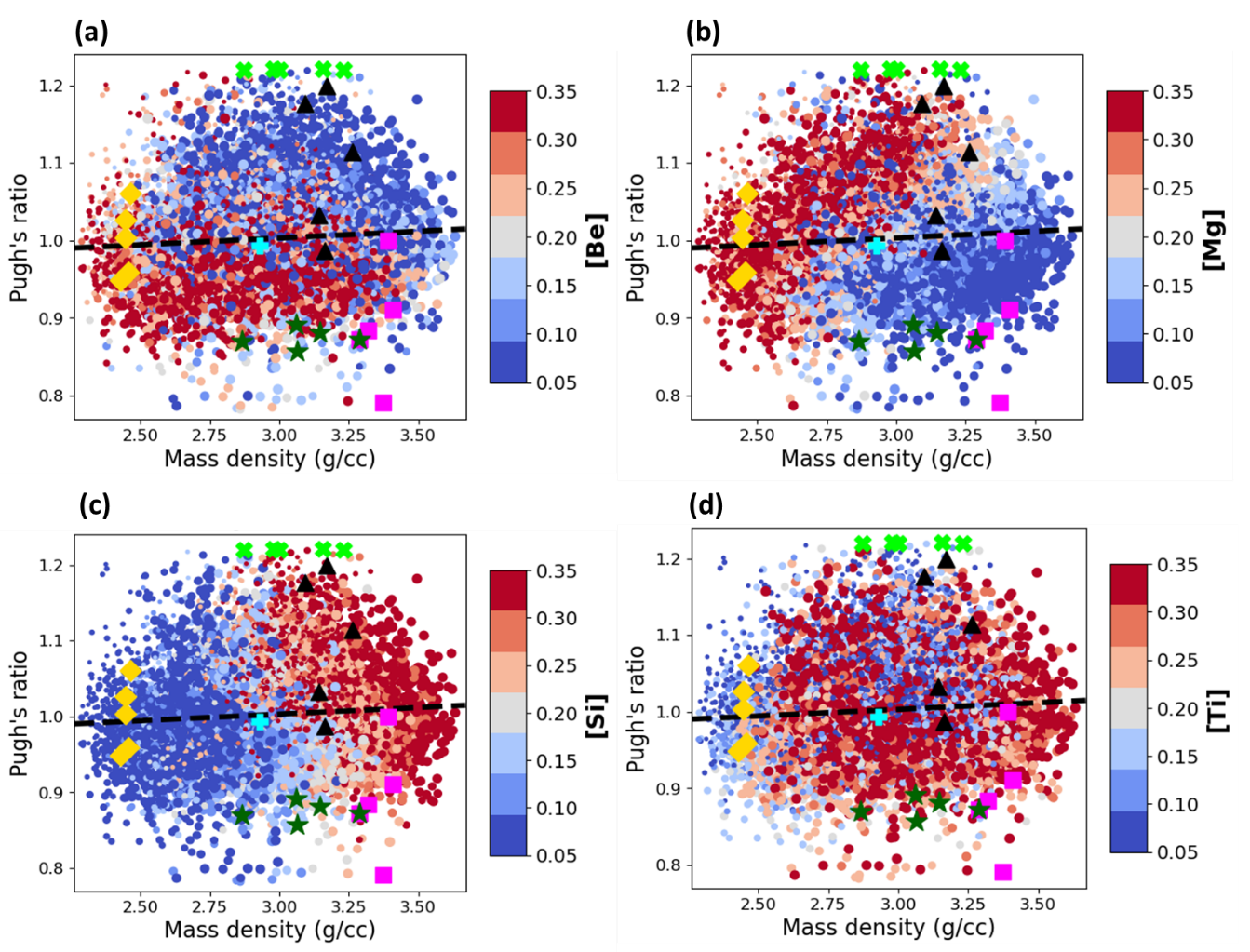


Figure S4: The Pugh’s ratio vs. the mass density for the compositions of AlBeMgTiSi with BCC lattice in SS phase. The black triangles correspond to the topmost energetically stable compositions, yellow diamonds represent the topmost stable compositions with the lowest density, and green stars indicate ones with the highest specific Young’s modulus. The magenta squares indicate the topmost stable compositions with the highest Young’s modulus and the topmost stable compositions with the highest Pugh’s ratio indicated by green crosses. The equimolar composition is specified by cyan plus marker. The dashed line indicates a weak correlation between the Pugh’s ratio and mass density. The marker color indicates the molar fraction of Be (a), Mg (b), Si (c) and Ti (d) as shown by the side colorbar, and the marker size is proportional to the molar fraction of Al.

Table S5: The stable compositions of AlBeMgTiSi with the highest Pugh’s ratio sorted in descending order according to their values. For each composition we report the formation energy, mass density, elastic modulus, and specific elastic modulus.

| **Composition** | **Pugh’s ratio (B/G)** | **Formation energy (eV/atom)** | **Mass density (g/cc)** | **Young’s modulus (GPa)** | **Specific stiffness (MN/kg)** |
| --- | --- | --- | --- | --- | --- |
| [0.28, 0.11, 0.24, 0.15, 0.22] | 1.222 | -0.031 | 2.46 | 132.94 | 54.13 |
| [0.21, 0.25, 0.25, 0.05, 0.24] | 1.221 | -0.003 | 2.43 | 135.77 | 55.90 |
| [0.27, 0.19, 0.25, 0.05, 0.24] | 1.220 | -0.011 | 2.45 | 132.23 | 54.04 |
| [0.24, 0.25, 0.22, 0.05, 0.24] | 1.218 | -0.004 | 2.45 | 134.29 | 54.87 |
| [0.29, 0.23, 0.22, 0.05, 0.21] | 1.217 | -0.027 | 2.46 | 124.58 | 50.58 |

## **AlBeMgTiLi**

### ***Formation energy***

Figure S5 shows the formation energy vs. mass density for all grid compositions of AlBeMgTiLi with BCC lattice in SS phase.


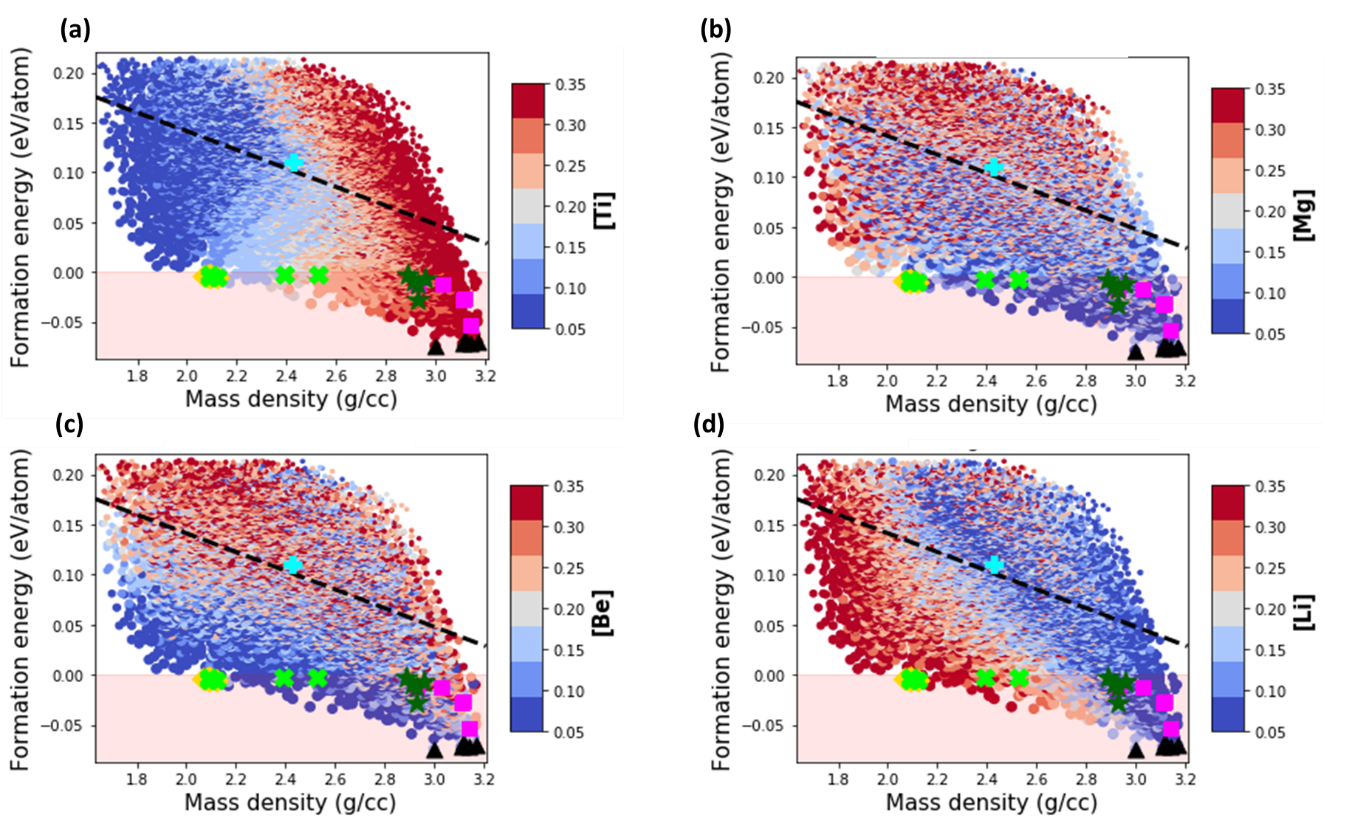


Figure S5: The formation energy per atom vs. the mass density for the compositions of AlBeMgTiLi with BCC lattice in SS phase. The black triangles correspond to the topmost energetically stable compositions, yellow diamonds represent the topmost stable compositions with the lowest density, and green stars indicate ones with the highest specific Young’s modulus. The magenta squares indicate the topmost stable compositions with the highest Young’s modulus and the topmost stable compositions with the highest Pugh’s ratio indicated by green crosses. The equimolar composition is specified by cyan plus marker. The dashed line indicates negative linear correlation between the formation energy and density. The marker color indicates the molar fraction of Ti (a), Mg (b), Be (c) and Li (d) as shown by the side colorbar, and the marker size is proportional to the molar fraction of Al.

Table S6: The five topmost energetically stable compositions of the selected AlBeMgTiLi sorted in ascending order according to their values. The mass density, elastic modulus, specific elastic modulus and Pugh’s ration are reported for each composition.

| **Composition** | **Energy (eV/atom)** | **Density (g/cc)** | **Elastic modulus (GPa)** | **Specific stiffness (MNm/kg)** | **Pugh’s ratio** |
| --- | --- | --- | --- | --- | --- |
| [0.35, 0.05, 0.14, 0.32, 0.14] | -0.074 | 3.01 | 156.4 | 52.05 | 1.28 |
| [0.35, 0.11, 0.11, 0.35, 0.08] | -0.072 | 3.14 | 161.27 | 51.28 | 1.28 |
| [0.35, 0.11, 0.08,0.35, 0.11] | -0.072 | 3.11 | 158.44 | 50.87 | 1.28 |
| [0.35, 0.23, 0.05, 0.32, 0.05] | -0.07 | 3.17 | 188.29 | 59.48 | 1.23 |
| [0.32, 0.17, 0.05, 0.35, 0.11] | -0.067 | 3.12 | 168.79 | 54.06 | 1.26 |

### ***Mass density***

The mean mass density of AlBeMgTiLi as a function of molar fraction of the constituent elements is shown in Figure S5.

Table S7: The most lightweight stable compositions of AlBeMgTiLi listed in an ascending order according to their values. In addition, we report the formation energy, elastic modulus, specific elastic modulus and Pugh’s ratio for each composition.

| **Composition** | **Density (g/cc)** | **Energy (eV/atom)** | **Elastic modulus (GPa)** | **Specific stiffness (MNm/kg)** | **Pugh’s ratio** |
| --- | --- | --- | --- | --- | --- |
| [0.35, 0.05, 0.14, 0.11, 0.35] | 2.07 | -0.004 | 88.88 | 43.02 | 1.26 |
| [0.32, 0.08, 0.17, 0.11, 0.32] | 2.08 | -0.004 | 91.18 | 43.94 | 1.24 |
| [0.35, 0.05, 0.29, 0.08, 0.23] | 2.09 | -0.005 | 83.48 | 40.03 | 1.39 |
| [0.35, 0.05, 0.32, 0.08, 0.2] | 2.09 | -0.002 | 86.49 | 41.3 | 1.36 |
| [0.35, 0.05, 0.35, 0.08, 0.17] | 2.12 | -0.006 | 86.05 | 40.62 | 1.37 |

### ***Elastic modulus***

Figure S6 shows the elastic modulus vs. mass density for all compositions of AlBeMgTiLi.


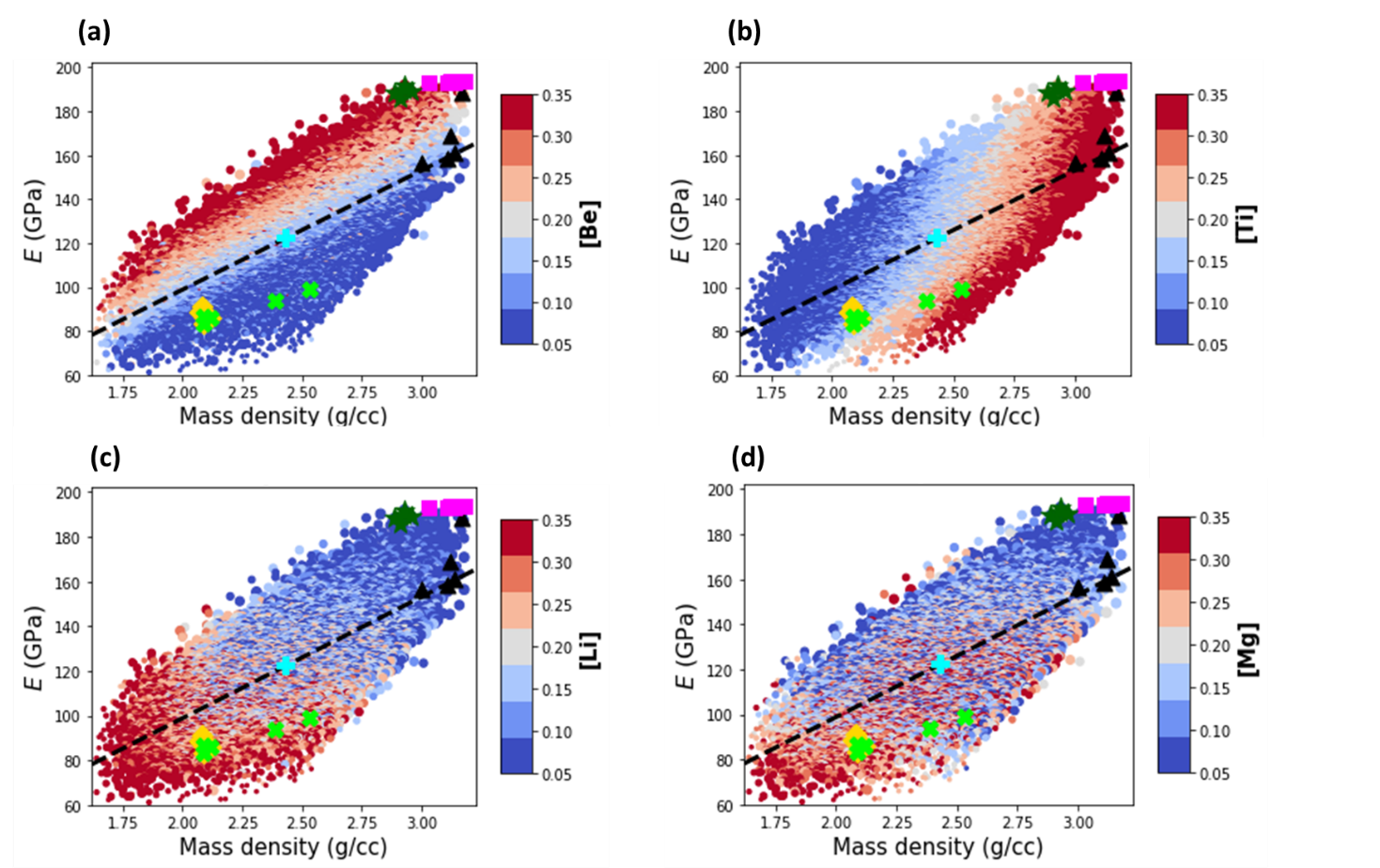


Figure S6: The elastic modulus vs. the mass density for the grid compositions of AlBeMgTiLi with BCC lattice in SS phase. The black triangles correspond to the topmost energetically stable compositions, yellow diamonds represent the topmost stable compositions with the lowest density, green stars indicate ones with the highest specific Young’s modulus. The magenta squares indicate the topmost stable compositions with the highest Young’s modulus and the topmost stable compositions with the highest Pugh’s ratio indicated by green crosses. The equimolar composition is specified by cyan plus marker. The dashed line indicates positive linear correlation between the Young’s modulus and mass density. The marker color indicates the molar fraction of Be (a), Ti (b), Li (c) and Mg (d) as shown by the side colorbar, and the marker size is proportional to the molar fraction of Al.

Table S8: The stable compositions of AlBeMgTiLi with the largest Young’s modulus sorted in descending order according to their values. For each composition, we report the formation energy, mass density, specific elastic modulus and Pugh’s ratio.

| **Composition** | **Elastic modulus (GPa)** | **Energy (eV/atom)** | **Density (g/cc)** | **Specific stiffness (MNm/kg)** | **Pugh’s ratio** |
| --- | --- | --- | --- | --- | --- |
| [0.26, 0.32, 0.05, 0.32, 0.05] | 193.45 | -0.027 | 3.12 | 62.05 | 1.21 |
| [0.25, 0.33, 0.05, 0.32, 0.05] | 193.36 | -0.028 | 3.11 | 62.25 | 1.21 |
| [0.26, 0.35, 0.05, 0.29, 0.05] | 193.25 | -0.013 | 3.03 | 63.68 | 1.17 |
| [0.29, 0.29, 0.05, 0.32, 0.05] | 193.11 | -0.054 | 3.14 | 61.55 | 1.19 |
| [0.33, 0.33, 0.05, 0.24, 0.05] | 192.06 | -0.012 | 2.93 | 65.53 | 1.18 |

### ***Specific elastic modulus***


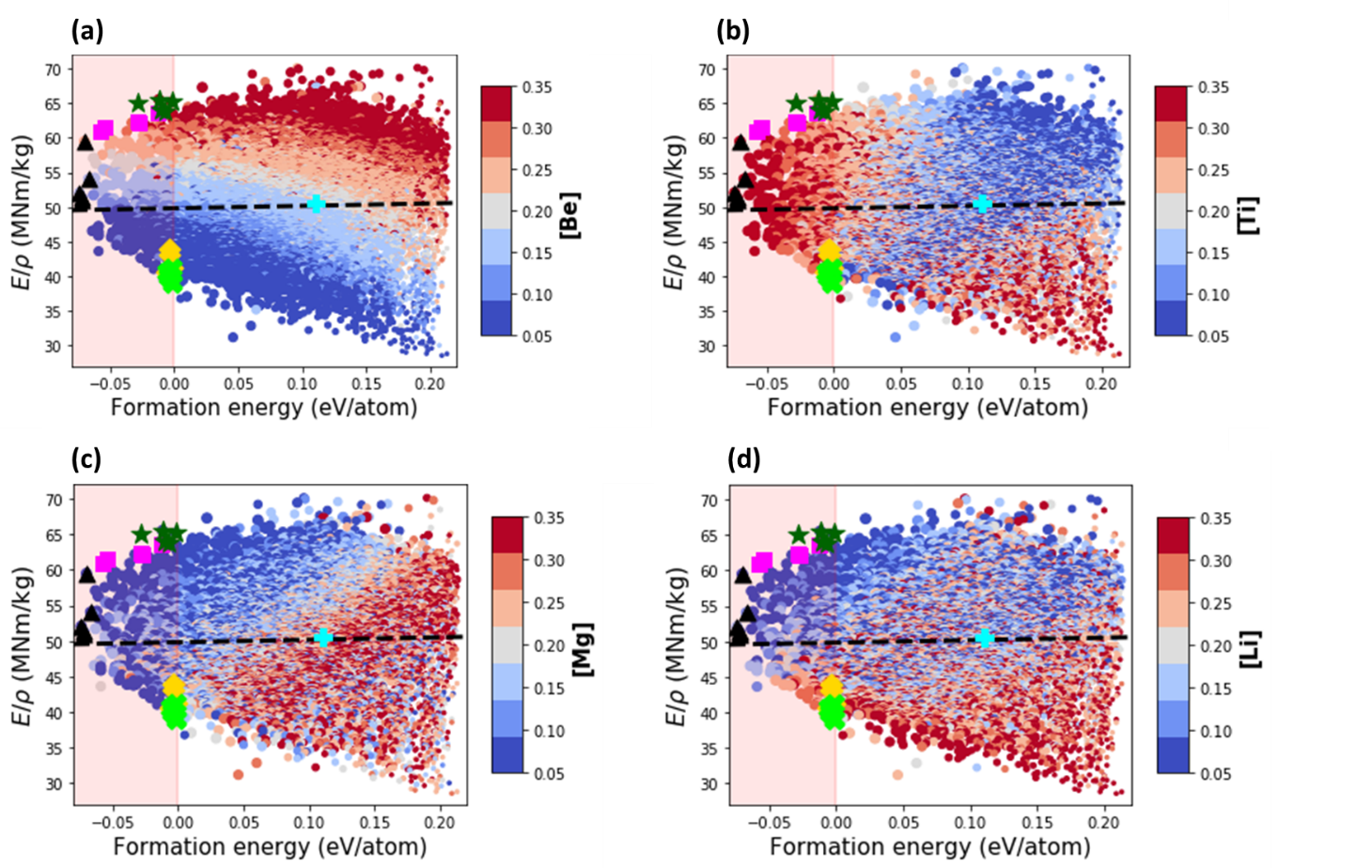


Figure S7: The specific elastic modulus vs. the formation energy per atom for the compositions of AlBeMgTiLi with BCC lattice in SS phase. The black triangles correspond to the topmost energetically stable compositions, yellow diamonds represent the topmost stable compositions with lowest density, green stars indicate ones with the highest specific Young’s modulus. The magenta squares indicate the topmost stable compositions with the highest Young’s modulus and the topmost stable compositions with the highest Pugh’s ratio indicated by green crosses. The equimolar composition is specified by cyan plus marker. The dashed line indicates positive correlation between the specific elastic modulus and formation energy. The marker color indicates the molar fraction of Be (a), Ti (b), Mg (c) and Li (d) as shown by the side colorbar, and the marker size is proportional to the molar fraction of Al.

Table S9: The stable compositions of AlBeMgTiLi with the largest specific elastic modulus sorted in descending order according to their values. For each composition, we report the formation energy, mass density, elastic modulus, and Pugh’s ratio.

| **Composition** | **Specific stiffness (MNm/kg)** | **Energy (eV/atom)** | **Density (g/cc)** | **Elastic modulus (GPa)** | **Pugh’s ratio** |
| --- | --- | --- | --- | --- | --- |
| [0.33, 0.33, 0.05, 0.24, 0.05] | 65.53 | -0.012 | 2.93 | 192.06 | 1.18 |
| [0.32, 0.35, 0.05, 0.23, 0.05] | 65.19 | -0.002 | 2.89 | 188.58 | 1.2 |
| [0.29, 0.25, 0.05, 0.34, 0.05] | 64.97 | -0.028 | 2.93 | 190.07 | 1.11 |
| [0.35, 0.32, 0.05, 0.23, 0.05] | 63.99 | -0.01 | 2.91 | 186.1 | 1.21 |
| [0.29, 0.35, 0.05, 0.26, 0.05] | 63.8 | -0.007 | 2.96 | 188.95 | 1.2 |

### ***Pugh’s ratio***


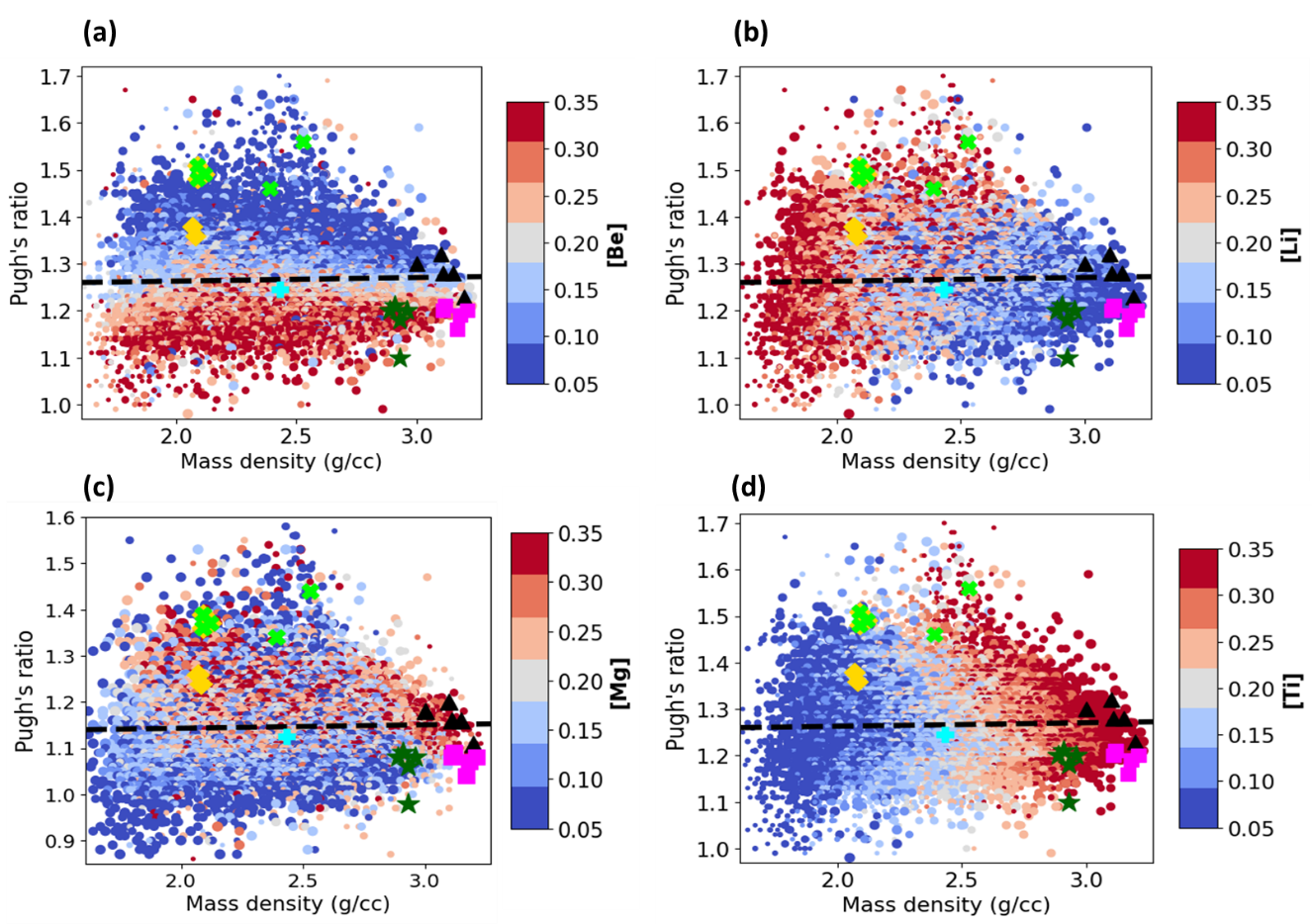


Figure S8: The Pugh’s ratio vs. the mass density for the compositions of AlBeMgTiLi with BCC lattice in SS phase. The black triangles correspond to the topmost energetically stable compositions, yellow diamonds represent the topmost stable compositions with the lowest density, green stars indicate ones with highest specific Young’s modulus. The magenta squares indicate the topmost stable compositions with the highest Young’s modulus and the topmost stable compositions with the highest Pugh’s ratio indicated by green crosses. The equimolar composition is specified by cyan plus marker. The dashed line indicates a weak correlation between the Pugh’s ratio and mass density. The marker color indicates the molar fraction of Be (a), Li (b), Mg (c), Ti (d) as shown by the side colorbar, and the marker size is proportional to the molar fraction of Al.

Table S10: The stable compositions of AlBeMgTiLi with the highest Pugh’s ratio sorted in descending order according to their values. For each composition, we report the formation energy, mass density, elastic modulus, and specific elastic modulus.

| **Composition** | **Pugh’s ratio** | **Energy (eV/atom)** | **Density (g/cc)** | **Elastic modulus (GPa)** | **Specific stiffness (MNm/kg)** |
| --- | --- | --- | --- | --- | --- |
| [0.32, 0.05, 0.17, 0.18, 0.28,] | 1.56 | -0.002 | 2.53 | 98.82 | 38.99 |
| [0.35, 0.05, 0.29, 0.08, 0.23] | 1.51 | -0.005 | 2.09 | 83.48 | 40.03 |
| [0.32, 0.05, 0.25, 0.16, 0.22] | 1.51 | -0.025 | 3.14 | 153.9 | 49.01 |
| [0.35, 0.05, 0.35, 0.08, 0.17] | 1.49 | -0.006 | 2.12 | 86.05 | 40.62 |
| [0.35, 0.05, 0.32, 0.08, 0.2] | 1.48 | -0.002 | 2.09 | 86.49 | 41.31 |

## **AlBeMgTiCu**

### ***Formation energy***


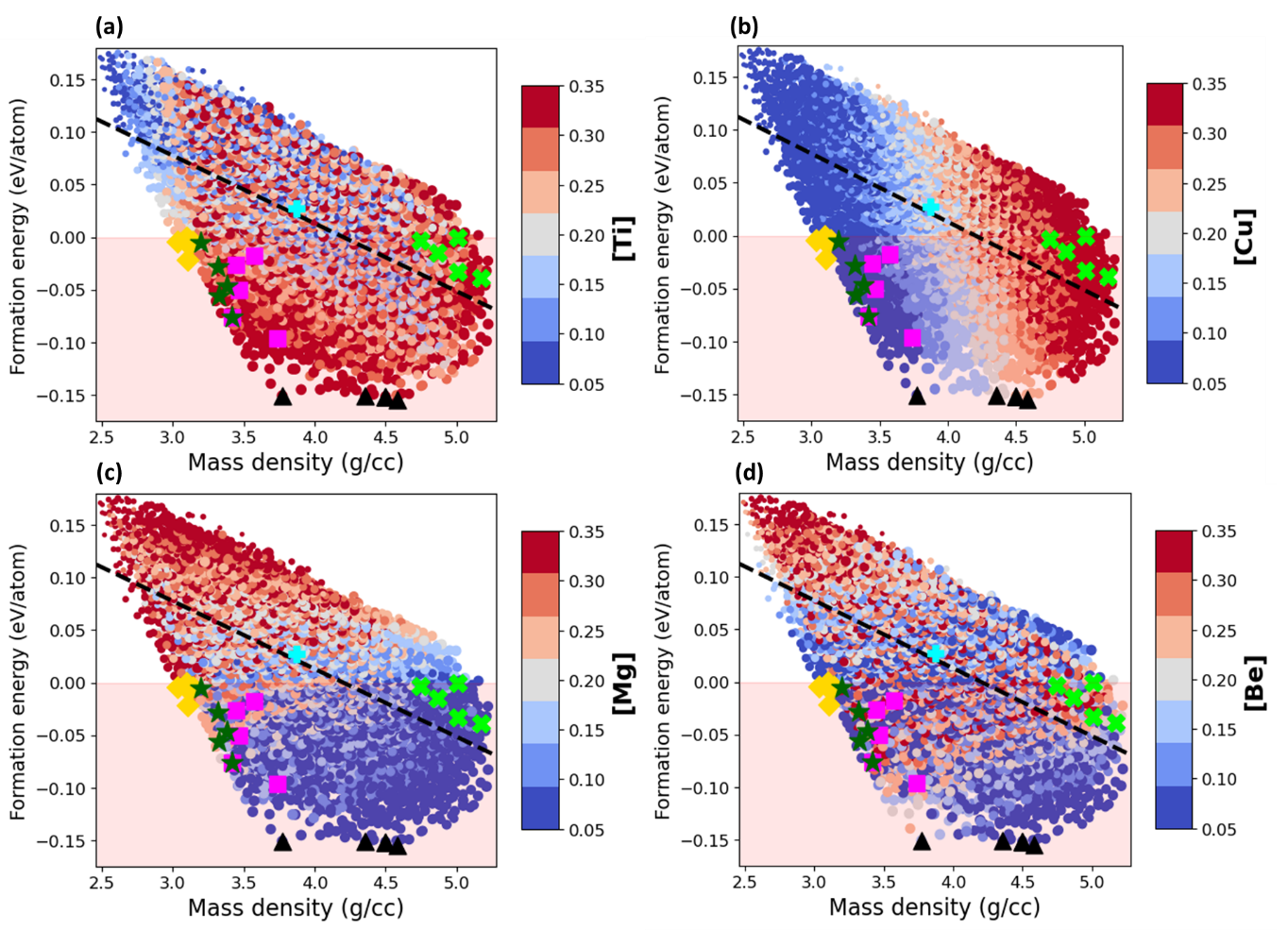


Figure S9: The formation energy per atom vs. the mass density for the compositions of AlBeMgTiCu with BCC lattice in SS phase. The black triangles correspond to the topmost energetically stable compositions, yellow diamonds represent the topmost stable compositions with the lowest density, and green stars indicate ones with the highest specific Young’s modulus. The magenta squares indicate the topmost stable compositions with the highest Young’s modulus and the topmost stable compositions with the highest Pugh’s ratio indicated by green crosses. The equimolar composition is specified by cyan plus marker. The dashed line indicates negative linear correlation between the formation energy and density. The marker color indicates the molar fraction of Ti (a), Cu (b), Mg (c) and Be (d) as shown by the side colorbar, and the marker size is proportional to the molar fraction of Al.

Table S11: The most lightweight stable compositions of AlBeMgTiCu listed in an ascending order according to their values. In addition, we report the formation energy, elastic modulus, specific elastic modulus and Pugh’s ratio for each composition.

| **Composition** | **Formation energy (eV/atom)** | **Mass density (g/cc)** | **Young’s modulus (GPa)** | **Specific Young’s modulus (MN/kg)** | **Pugh’s ratio (B/G)** |
| --- | --- | --- | --- | --- | --- |
| [0.32, 0.05, 0.05, 0.35, 0.23] | -0.155 | 4.58 | 166.73 | 36.40 | 1.33 |
| [0.35, 0.05, 0.05, 0.32, 0.23] | -0.152 | 4.50 | 172.06 | 38.24 | 1.29 |
| [0.35, 0.05, 0.05, 0.3, 0.25] | -0.152 | 4.50 | 172.06 | 38.24 | 1.29 |
| [0.35, 0.08, 0.05, 0.32, 0.2] | -0.152 | 4.35 | 171.43 | 39.37 | 1.27 |
| [0.35, 0.08, 0.05, 0.35, 0.17] | -0.151 | 3.77 | 190.11 | 50.42 | 1.27 |

### ***Mass density***

Table S12: The most lightweight stable compositions of AlBeMgTiCu listed in an ascending order according to their values. In addition, we report the formation energy, elastic modulus, specific elastic modulus and Pugh’s ratio for each composition.

| **Composition** | **Mass density (g/cc)** | **Formation energy (eV/atom)** | **Young’s modulus (GPa)** | **Specific Young’s modulus (MN/kg)** | **Pugh’s ratio (B/G)** |
| --- | --- | --- | --- | --- | --- |
| [0.35, 0.08, 0.29, 0.23, 0.05] | 3.03 | -0.004 | 144.57 | 47.65 | 1.30 |
| [0.32, 0.08, 0.29, 0.26, 0.05] | 3.09 | -0.004 | 138.13 | 44.67 | 1.31 |
| [0.29, 0.05, 0.33, 0.28, 0.05] | 3.09 | 0.000 | 133.00 | 42.99 | 1.30 |
| [0.35, 0.05, 0.29, 0.26, 0.05] | 3.11 | -0.022 | 142.38 | 45.83 | 1.27 |
| [0.35, 0.17, 0.2, 0.23, 0.05] | 3.14 | -0.004 | 142.40 | 45.34 | 1.32 |

### ***Elastic modulus***


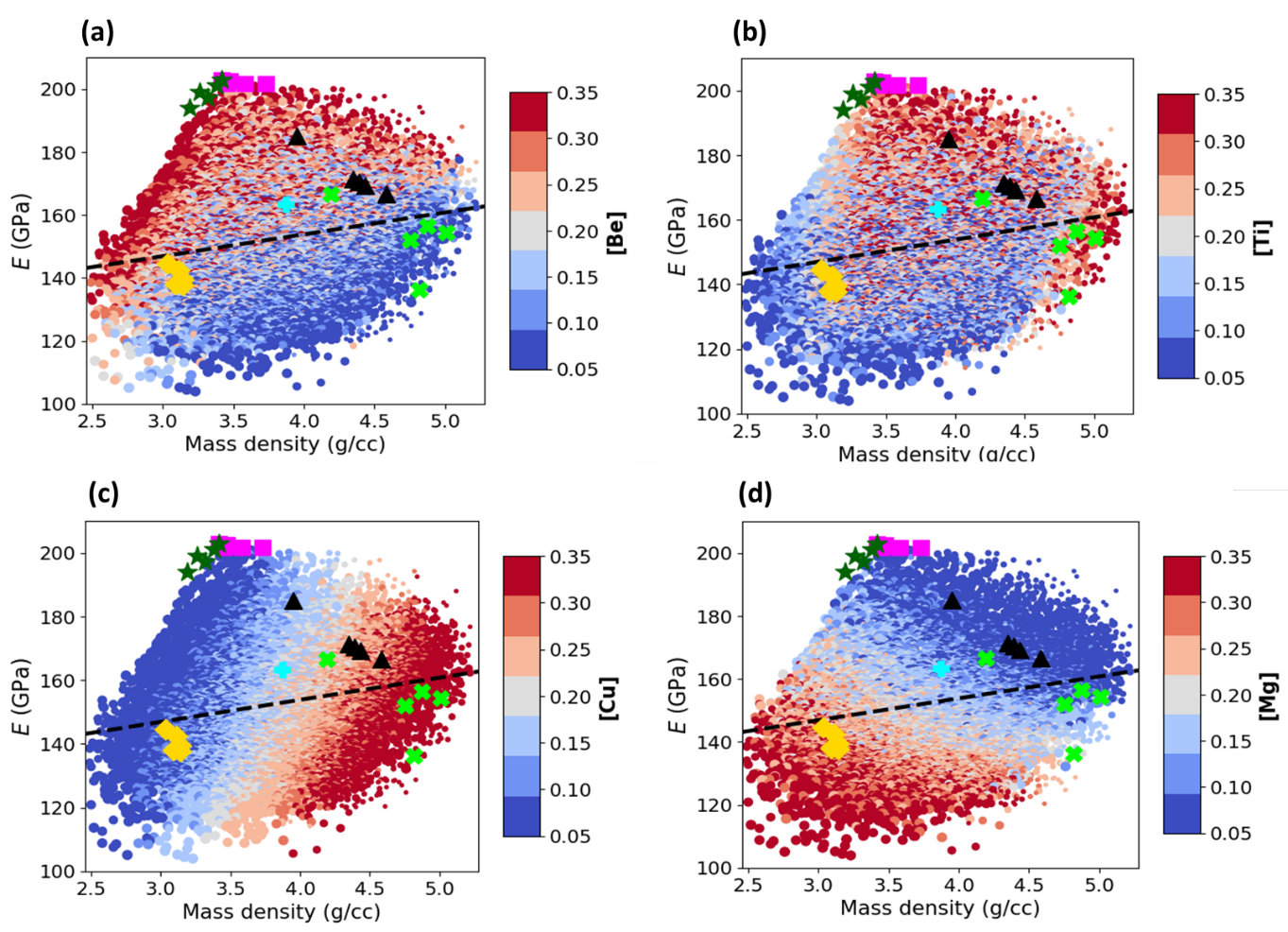


Figure S10: The elastic modulus vs. the mass density for the grid compositions of AlBeMgTiCu with BCC lattice in SS phase. The black triangles correspond to the topmost energetically stable compositions, yellow diamonds represent the topmost stable compositions with the lowest density, and green stars indicate ones with the highest specific Young’s modulus. The magenta squares indicate the topmost stable compositions with the highest Young’s modulus and the topmost stable compositions with the highest Pugh’s ratio indicated by green crosses. The equimolar composition is specified by cyan plus marker. The dashed line indicates positive linear correlation between the Young’s modulus and mass density. The marker color indicates the molar fraction of Be (a), Ti (b), Cu (c) and Mg (d) as shown by the side colorbar, and the marker size is proportional to the molar fraction of Al.

Table S13: The stable compositions of AlBeMgTiCu with the largest Young’s modulus sorted in descending order according to their values. For each composition, we report the formation energy, mass density, specific elastic modulus and Pugh’s ratio.

| **Composition** | **Young’s modulus (GPa)** | **Formation energy (eV/atom)** | **Mass density (g/cc)** | **Specific Young’s modulus (MN/kg)** | **Pugh’s ratio (B/G)** |
| --- | --- | --- | --- | --- | --- |
| [0.25, 0.32, 0.05, 0.32, 0.05] | 202.93 | -0.076 | 3.42 | 59.40 | 1.19 |
| [0.32, 0.32, 0.05, 0.26, 0.05] | 202.46 | -0.051 | 3.47 | 58.42 | 1.20 |
| [0.21, 0.33, 0.09, 0.32, 0.05] | 201.81 | -0.027 | 3.45 | 58.56 | 1.19 |
| [0.2, 0.35, 0.08, 0.29, 0.08] | 201.73 | -0.018 | 3.58 | 56.41 | 1.17 |
| [0.29, 0.25, 0.05, 0.32, 0.09] | 201.61 | -0.096 | 3.73 | 53.98 | 1.20 |

### ***Specific elastic modulus***


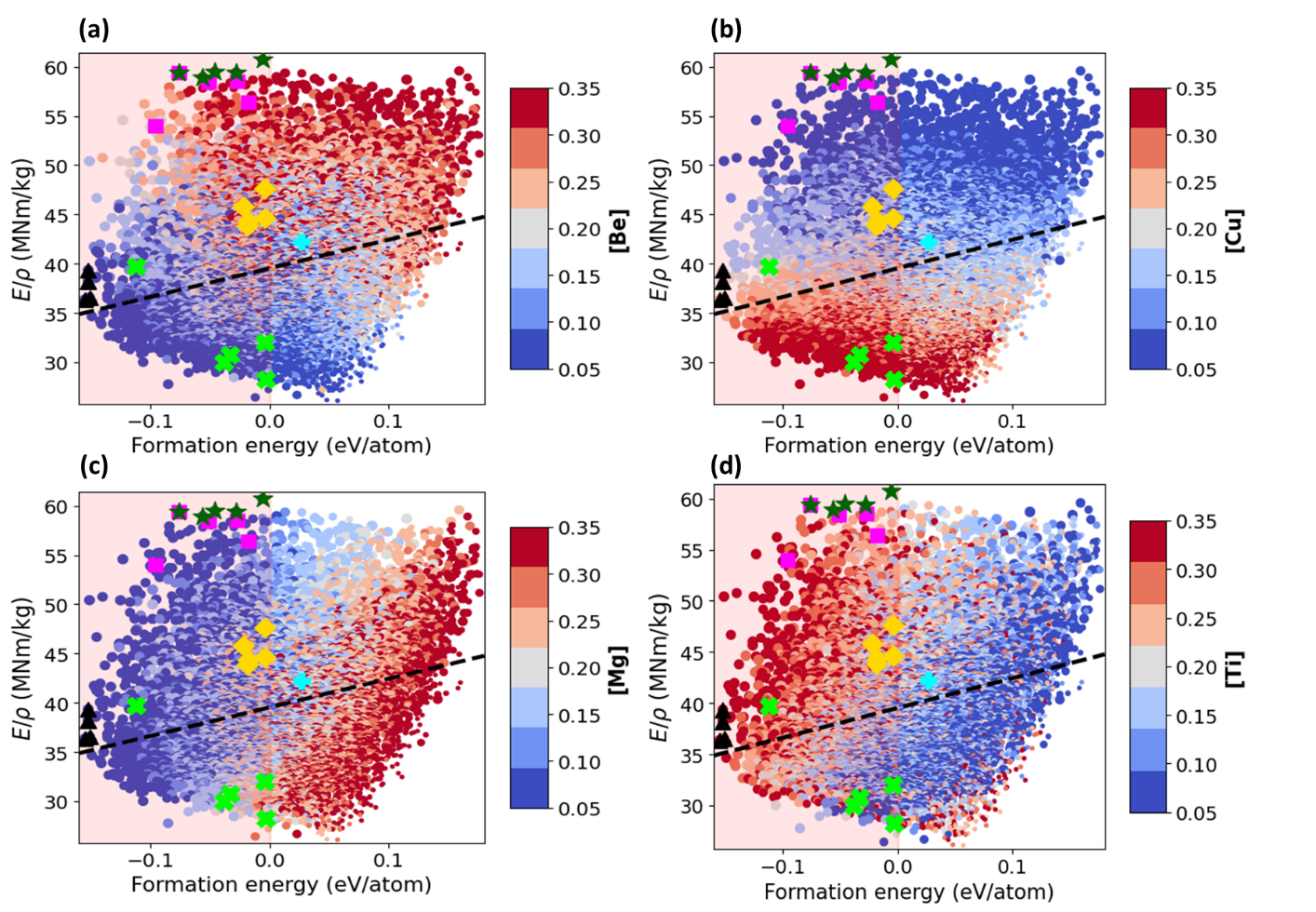


Figure S11: The specific elastic modulus vs. the formation energy per atom for the compositions of AlBeMgTiCu with BCC lattice in SS phase. The black triangles correspond to the topmost energetically stable compositions, yellow diamonds represent the topmost stable compositions with the lowest density, and green stars indicate ones with the highest specific Young’s modulus. The magenta squares indicate the topmost stable compositions with the highest Young’s modulus and the topmost stable compositions with the highest Pugh’s ratio indicated by green crosses. The equimolar composition is specified by cyan plus marker. The dashed line indicates positive correlation between the specific elastic modulus and formation energy. The marker color indicates the molar fraction of Be (a), Cu (b), Mg (c) and Ti (d) as shown by the side colorbar, and the marker size is proportional to the molar fraction of Al.

Table S14: The stable compositions of AlBeMgTiCu with the largest specific elastic modulus sorted in descending order according to their values. For each composition, we report the formation energy, mass density, elastic modulus, and Pugh’s ratio.

| **Composition** | **Specific stiffness (MN/kg)** | **Formation energy (eV/atom)** | **Mass density (g/cc)** | **Young’s modulus (GPa)** | **Pugh’s ratio (B/G)** |
| --- | --- | --- | --- | --- | --- |
| [0.32, 0.35, 0.08, 0.2, 0.05] | 60.71 | -0.006 | 3.19 | 193.96 | 1.17 |
| [0.29, 0.35, 0.05, 0.26, 0.05] | 59.52 | -0.046 | 3.38 | 201.04 | 1.19 |
| [0.32, 0.35, 0.05, 0.23, 0.05] | 59.43 | -0.028 | 3.32 | 197.10 | 1.23 |
| [0.32, 0.32, 0.05, 0.26, 0.05] | 59.40 | -0.076 | 3.42 | 202.93 | 1.19 |
| [0.33, 0.33, 0.05, 0.24, 0.05] | 58.95 | -0.056 | 3.33 | 196.10 | 1.21 |

### ***Pugh’s ratio***


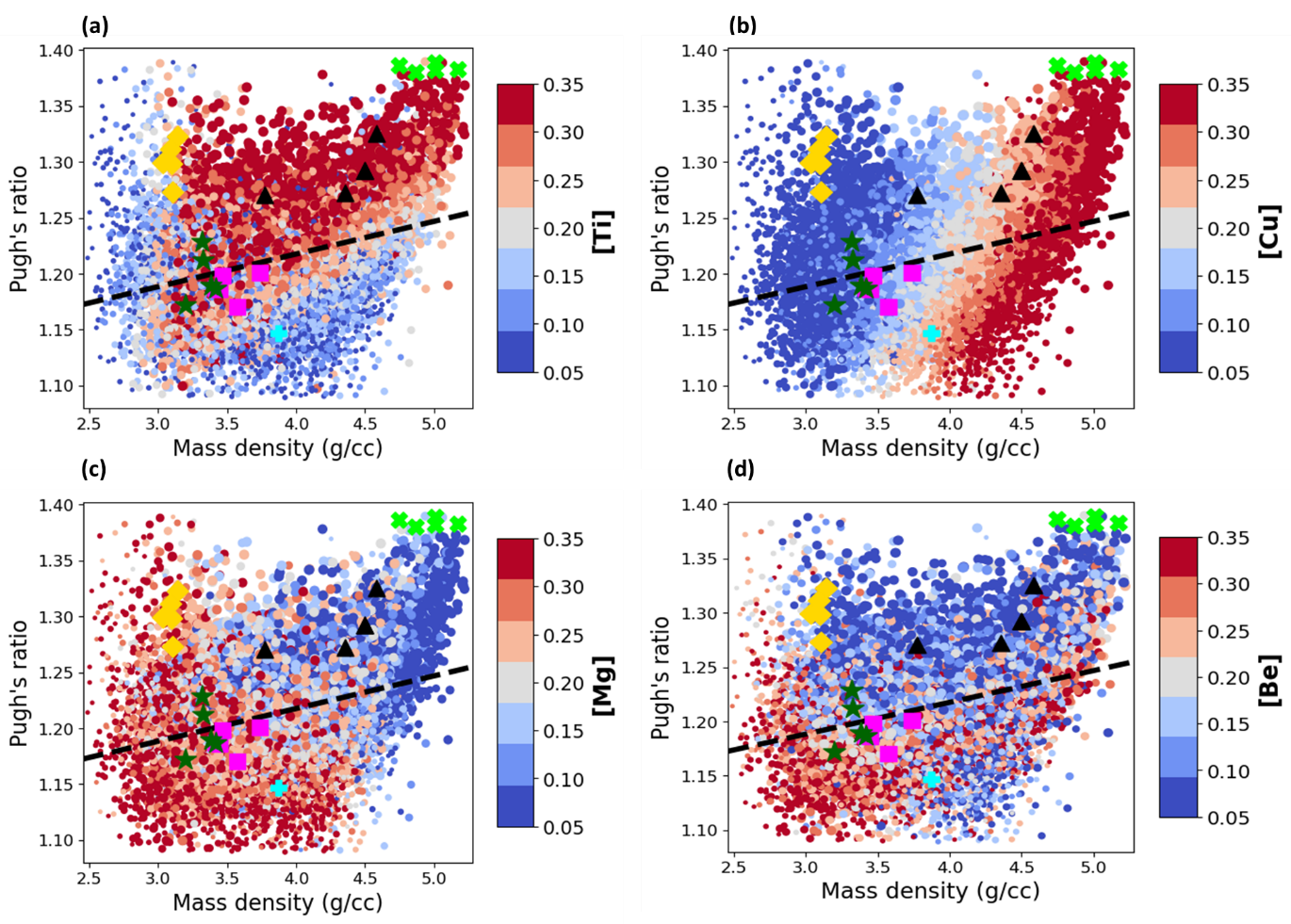


Figure S12: The Pugh’s ratio vs. the mass density for the compositions of AlBeMgTiCu with BCC lattice in SS phase. The black triangles correspond to the topmost energetically stable compositions, yellow diamonds represent the topmost stable compositions with the lowest density, and green stars indicate ones with the highest specific Young’s modulus. The magenta squares indicate the topmost stable compositions with the highest Young’s modulus and the topmost stable compositions with the highest Pugh’s ratio indicated by green crosses. The equimolar composition is specified by cyan plus marker. The dashed line indicates a weak correlation between the Pugh’s ratio and mass density. The marker color indicates the molar fraction of Ti (a), Cu (b), Mg (c) and Be (d) as shown by the side colorbar, and the marker size is proportional to the molar fraction of Al.

Table S15: The stable compositions of AlBeMgTiCu with the highest Pugh’s ratio sorted in descending order according to their values. For each composition, we report the formation energy, mass density, elastic modulus, and specific elastic modulus.

| **Composition** | **Pugh’s ratio (B/G)** | **Formation energy (eV/atom)** | **Mass density (g/cc)** | **Young’s modulus (GPa)** | **Specific stiffness (MN/kg)** |
| --- | --- | --- | --- | --- | --- |
| [0.13, 0.06, 0.14, 0.32, 0.35] | 1.39 | -0.004 | 5.01 | 141.74 | 28.29 |
| [0.08, 0.2, 0.11, 0.32, 0.29] | 1.39 | -0.004 | 4.75 | 152.12 | 32.05 |
| [0.11, 0.11, 0.08, 0.35, 0.35] | 1.38 | -0.038 | 5.17 | 155.22 | 30.02 |
| [0.11, 0.14, 0.08, 0.35, 0.3] | 1.38 | -0.033 | 5.01 | 154.33 | 30.80 |
| [0.08, 0.2, 0.08, 0.35, 0.29] | 1.38 | -0.015 | 4.87 | 156.61 | 32.18 |

# Substitution of the constituent elements


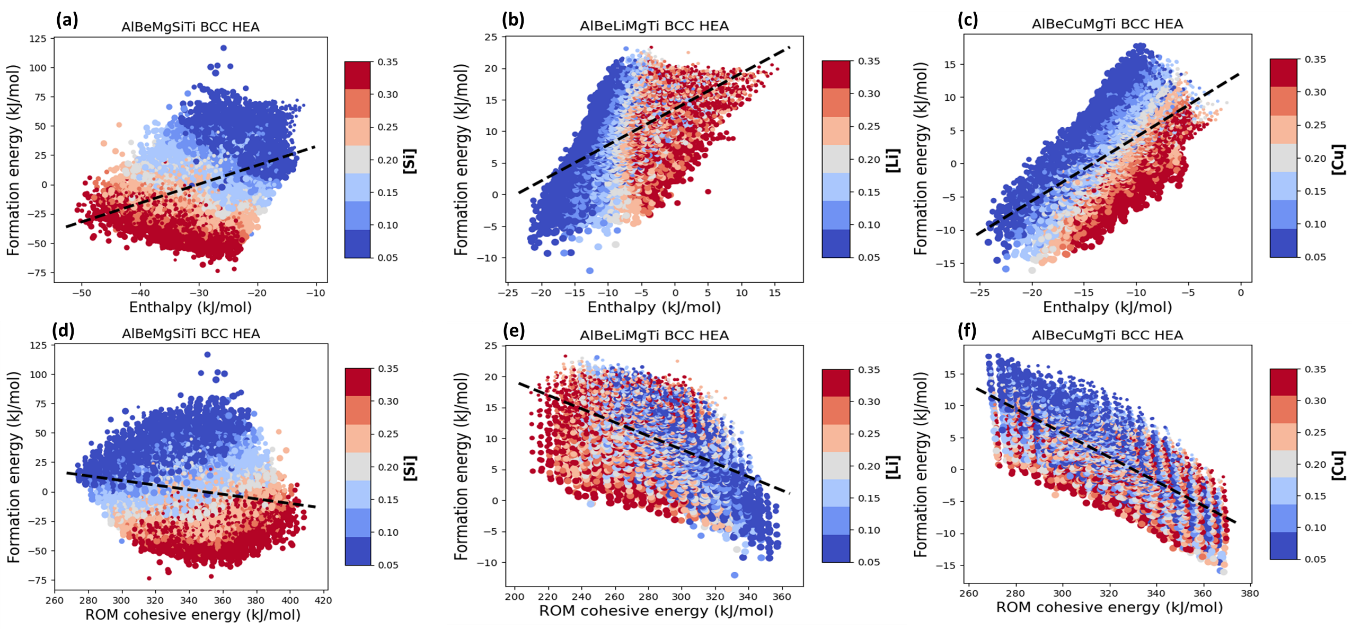


Figure S13: (a-c) The formation energy vs. the enthalpy of mixing for AlBeMgTiSi (a), AlBeMgTiLi (b), and AlBeMgTiCu (c). (d-f) The formation energy vs. the cohesive energy for AlBeMgTiSi (d), AlBeMgTiLi (e), and AlBeMgTiCu (f). The marker color indicates the molar fraction of the corresponding constituent elements as shown by the side colorbar and the marker size is proportional to the molar fraction of Al.


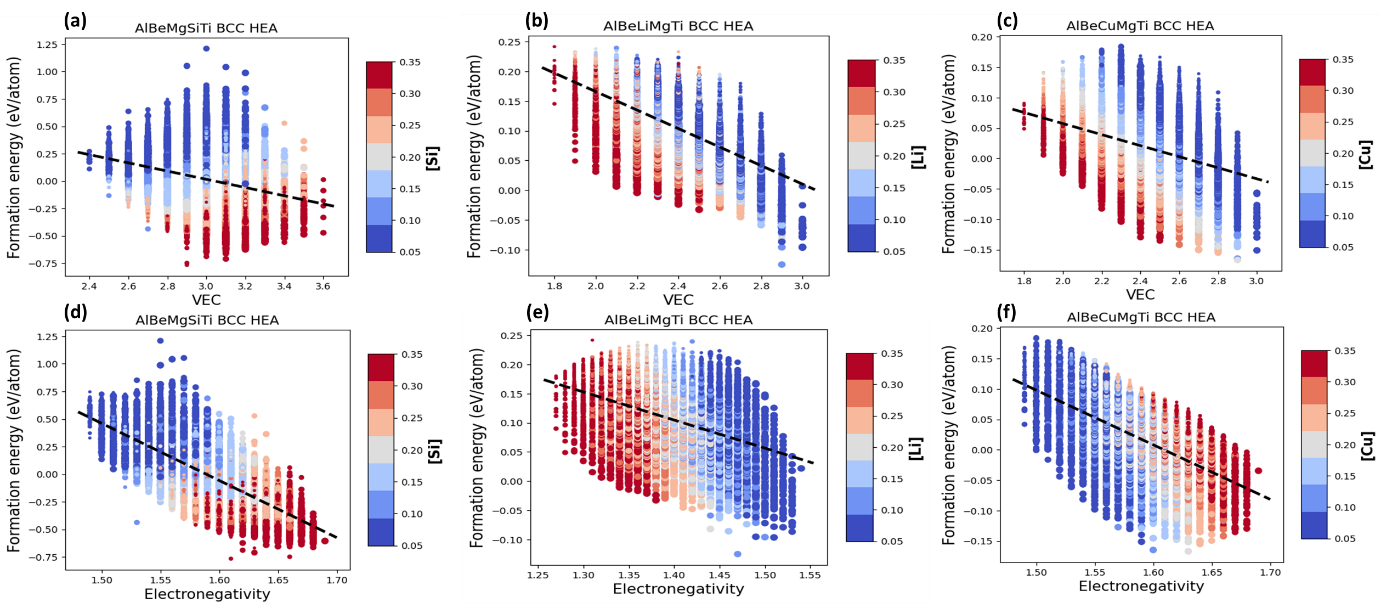


Figure S14: (a-c) The formation energy vs. VEC for AlBeMgTiSi (a), AlBeMgTiLi (b), and AlBeMgTiCu (c). (d-f) The formation energy vs. the Pauling electronegativity for AlBeMgTiSi (d), AlBeMgTiLi (e), and AlBeMgTiCu (f). The marker color indicates the molar fraction of the corresponding constituent elements as shown by the side colorbar and the marker size is proportional to the molar fraction of Al.


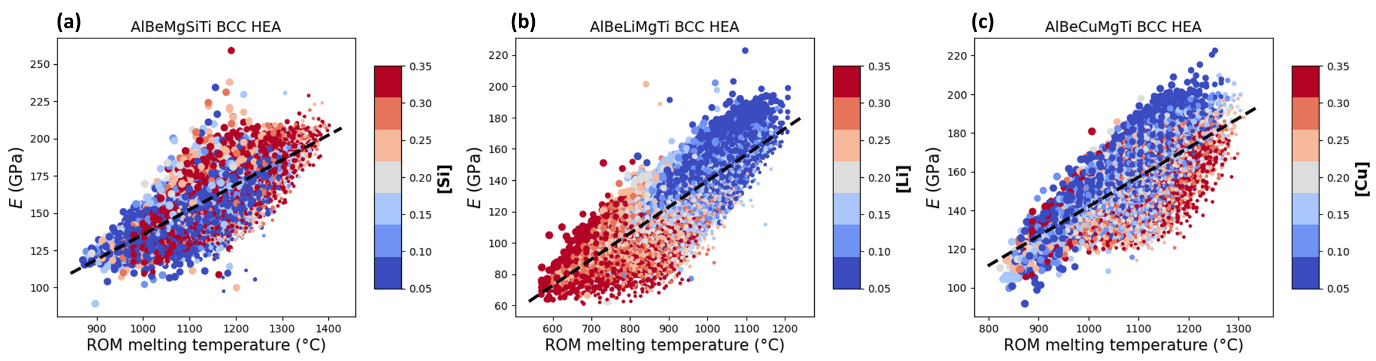


Figure S15: (a-c) The Young’s modulus vs. the melting temperature estimated by the rule of mixtures for AlBeMgTiSi (a), AlBeMgTiLi (b), and AlBeMgTiCu (c). The marker color indicates the molar fraction of the corresponding constituent elements as shown by the side colorbar and the marker size is proportional to the molar fraction of Al.


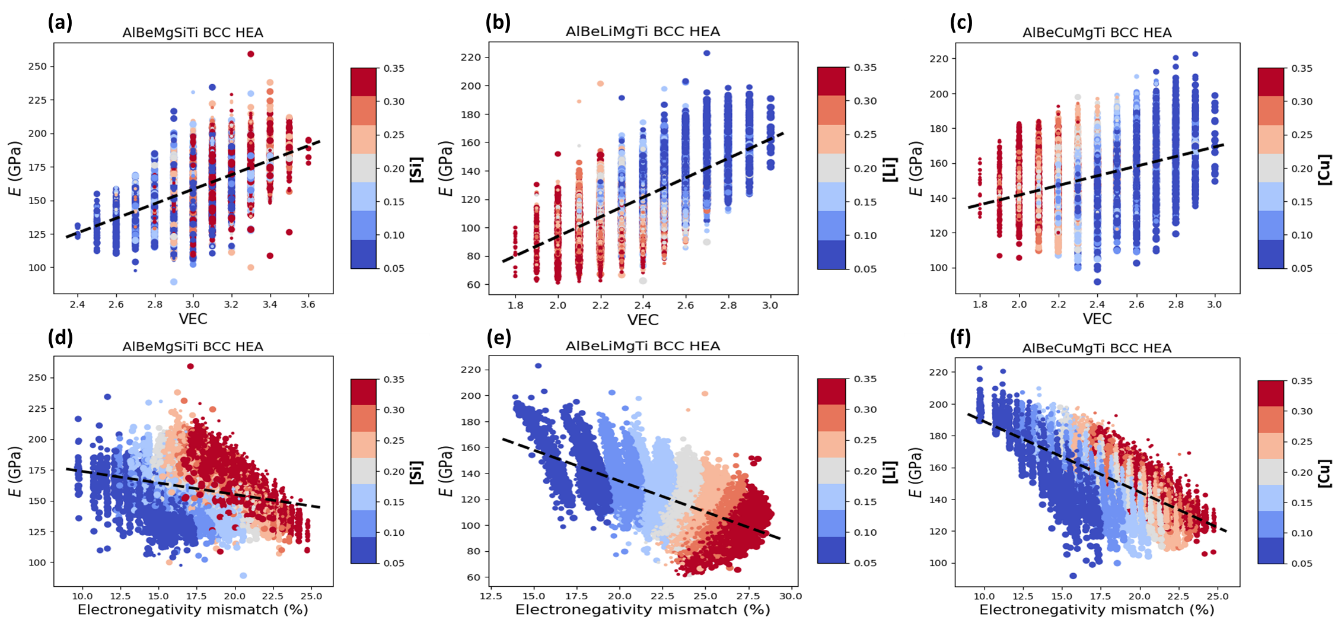


Figure S16: (a-c) The Young’s modulus vs. VEC for AlBeMgTiSi (a), AlBeMgTiLi (b), and AlBeMgTiCu (c). (d-f) The Young’s modulus vs. the Pauling electronegativity mismatch for AlBeMgTiSi (d), AlBeMgTiLi (e), and AlBeMgTiCu (f). The marker color indicates the molar fraction of the corresponding constituent elements as shown by the side colorbar and the marker size is proportional to the molar fraction of Al.


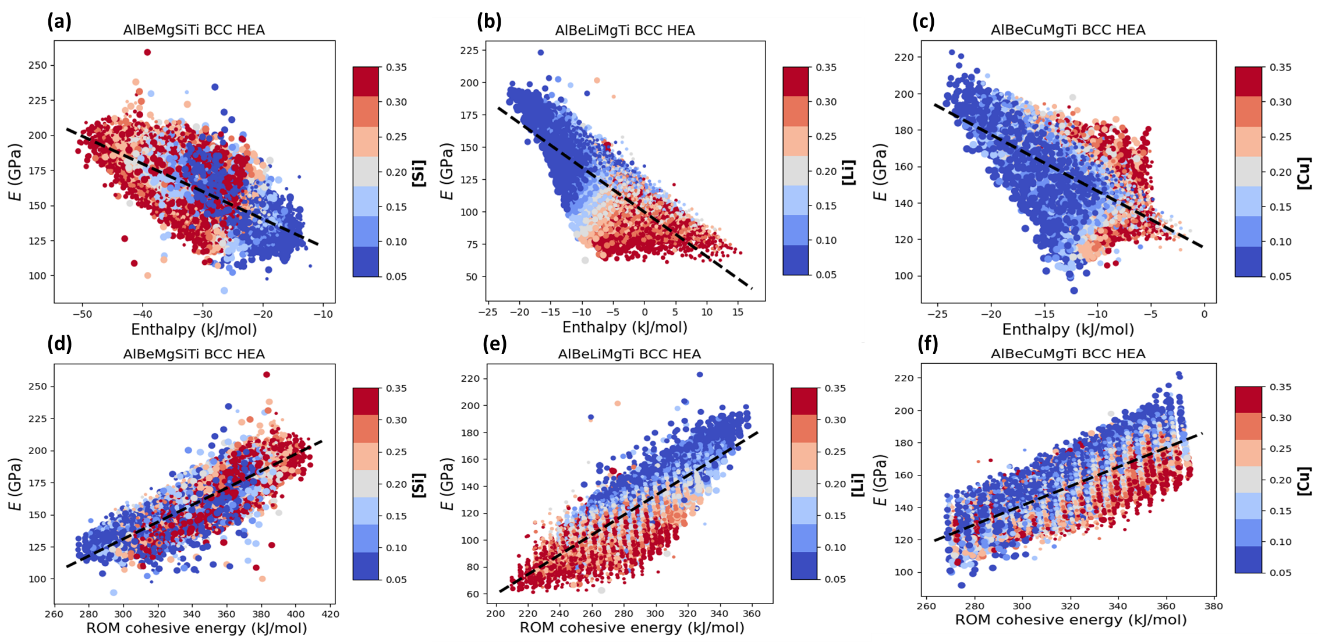


Figure S17: (a-c) The Young’s modulus vs. the enthalpy of mixing for AlBeMgTiSi (a), AlBeMgTiLi (b), and AlBeMgTiCu (c). (d-f) The Young’s modulus vs. cohesive energy for AlBeMgTiSi (d), AlBeMgTiLi (e), and AlBeMgTiCu (f). The marker color indicates the molar fraction of the corresponding constituent elements as shown by the side colorbar and the marker size is proportional to the molar fraction of Al.


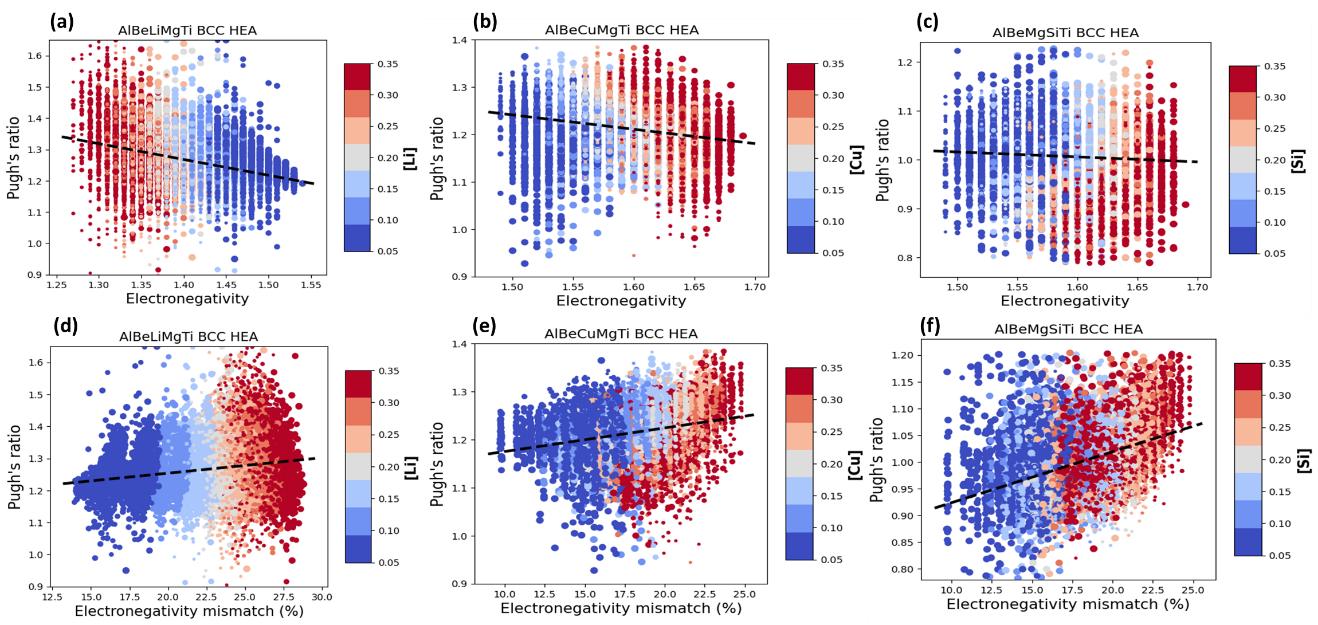


Figure S18: (a-c) The Pugh’s ratio vs. the Pauling electronegativity for AlBeMgTiLi (a), AlBeMgTiCu (b), and AlBeMgTiSi (c). (d-f) The Pugh’s ratio vs. the Pauling electronegativity mismatch for AlBeMgTiLi (d), AlBeMgTiCu (e), and AlBeMgTiSi (f). The marker color indicates the molar fraction of the corresponding constituent elements as shown by the side colorbar and the marker size is proportional to the molar fraction of Al.
